# Synthesis of novel purpurealidin analogs and evaluation of their effect on the cancer-relevant potassium channel K_V_10.1

**DOI:** 10.1371/journal.pone.0188811

**Published:** 2017-12-08

**Authors:** Lien Moreels, Chinmay Bhat, Manuela Voráčová, Steve Peigneur, Hannah Goovaerts, Eero Mäki-Lohiluoma, Farrah Zahed, Luis A. Pardo, Jari Yli-Kauhaluoma, Paula Kiuru, Jan Tytgat

**Affiliations:** 1 Toxicology and Pharmacology, Department of Pharmaceutical and Pharmacological Sciences, University of Leuven (KU Leuven), Leuven, Belgium; 2 Drug Research Program, Division of Pharmaceutical Chemistry and Technology, Faculty of Pharmacy, University of Helsinki, Helsinki, Finland; 3 Oncophysiology Group, Max-Planck-Institute of Experimental Medicine, Göttingen, Germany; University of Hull, UNITED KINGDOM

## Abstract

In the search for novel anticancer drugs, the potassium channel K_V_10.1 has emerged as an interesting cancer target. Here, we report a new group of K_V_10.1 inhibitors, namely the purpurealidin analogs. These alkaloids are produced by the Verongida sponges and are known for their wide variety of bioactivities. In this study, we describe the synthesis and characterization of 27 purpurealidin analogs. Structurally, bromine substituents at the central phenyl ring and a methoxy group at the distal phenyl ring seem to enhance the activity on K_V_10.1. The mechanism of action of the most potent analog **5** was investigated. A shift of the activation curve to more negative potentials and an apparent inactivation was observed. Since K_V_10.1 inhibitors can be interesting anticancer drug lead compounds, the effect of **5** was evaluated on cancerous and non-cancerous cell lines. Compound **5** showed to be cytotoxic and appeared to induce apoptosis in all the evaluated cell lines.

## Introduction

Although many efforts have been made to prevent and treat cancer, it is still one of the leading causes of death worldwide, with 8.8 million cancer deaths in 2015 [1]. A targeted approach as used in precision or personalized medicine could enhance the specificity of the treatment and minimize the negative side effects. The voltage-gated potassium channel human ether à go-go 1 (hEag1, K_V_10.1) represents an interesting cancer target because of its ectopic expression in over 70% of human cancers [2]. Moreover, transfection of rat Eag1 into mammalian cells induced features that are characteristic for malignant cell transformation [3]. K_V_10.1 inhibitors are considered to be lead compounds in the development of novel anticancer drugs [2]. In order to identify novel K_V_10.1 inhibitors or modulators, the effect of synthetic bromotyramine alkaloids on K_V_10.1-expressing oocytes was electrophysiologically investigated.

Bromotyrosine alkaloids are a large group of marine sponge metabolites mainly from the order Verongida, found at the coasts of Southeast Asia, Oceania, Japan and China [4–7]. Sponges have already shown to be a very fertile source of new toxins as they contain many secondary metabolites [8,9]. They have defensive, antibiotic, antiangiogenic, antiproliferative, hemolytic and cytotoxic properties. They inhibit mitosis and the assembly of microtubuli and they induce cytotoxic cell death [9]. In this way, metabolites that induce apoptosis might have potential as anticancer drugs [10]. The most striking sponge-derived compounds are the nucleosides spongothymidine and spongouridine, isolated from the *Tectitethya crypta*. They were the first marine derived compounds that were developed into a pharmaceutical drug. A derivative of these nucleosides is cytarabine (AraC), that is currently used as an anticancer agent in the treatment of leukemia [9–11].

**Fig 1:**
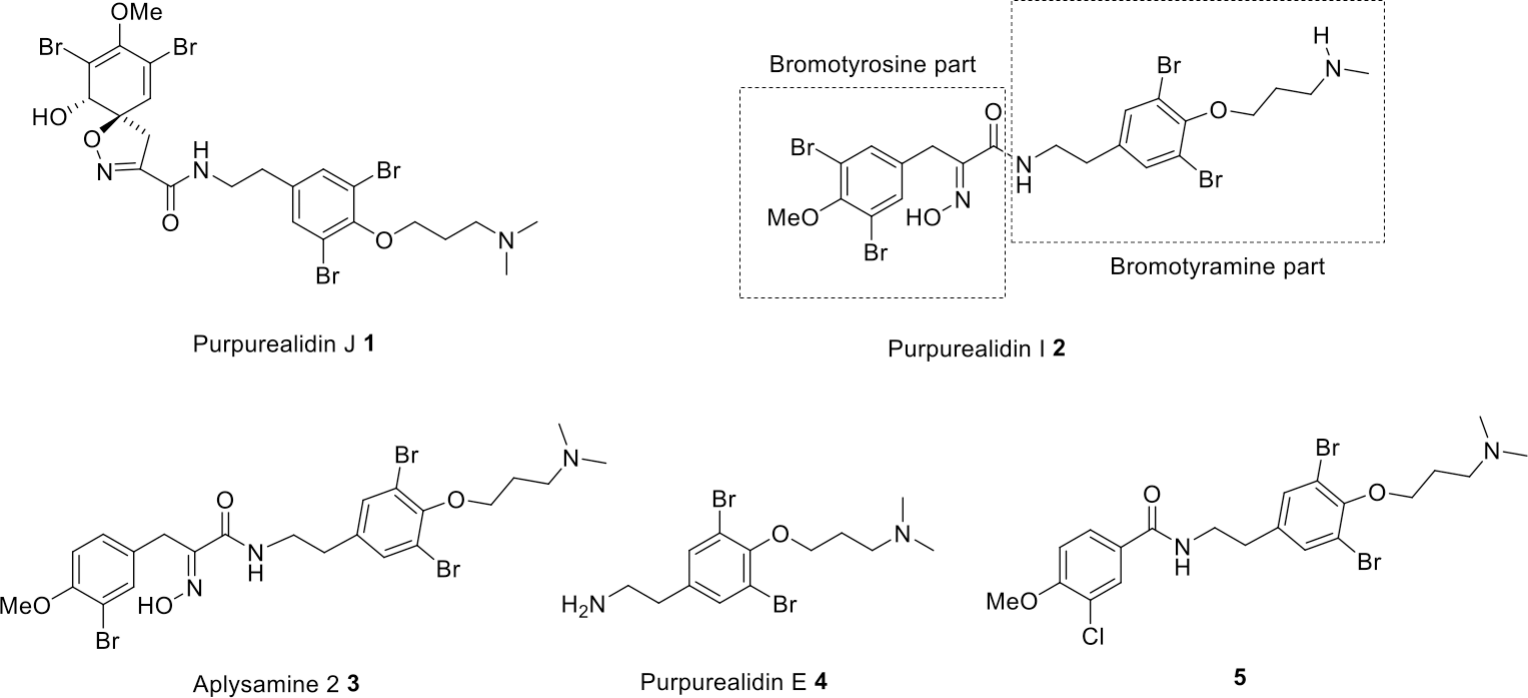
Marine bromotyrosines 1–4 and the most potent K_V_10.1 modulator analog 5.

A novel bromotyrosine purpurealidin J **1** (Fig 1) was found among other bromotyrosines (e.g. purpurealidin I **2**, aplysamine 2 **3**) in sponge *Pseudoceratina (Psammaplysilla) purpurea* by Tilvi and D’Souza [12]. These bromotyrosines acted as an inspiration for the syntheses of simplified amide analogs using bromotyramine purpurealidin E **4** as an amine starting material. As the nomenclature of the bromotyrosines is quite heterogeneous [6], we refer to our synthetic compounds as purpurealidin analogs. The effect of several synthetic analogs of these marine metabolites on K_V_10.1 was investigated. Several simplified purpurealidin analogs were identified to be K_V_10.1 modulators. The purpurealidin analog **5** (Fig 1) was found to be the most potent one and its effect on K_V_10.1-expressing oocytes and on various cancer and non-cancerous mammalian cell lines was investigated.

## Materials and methods

### Large scale synthesis of analog 5

All reactions were carried out using commercially available starting materials unless otherwise stated. The melting points were measured with Stuart SMP40 automated melting point apparatus and are uncorrected. ^1^H NMR (300 MHz) and ^13^C NMR (75 MHz) spectra in CDCl_3_ or *d*_6_-DMSO at ambient temperature were recorded on a Varian Mercury *Plus* 300 spectrometer. Chemical shifts (*δ*) are given in parts per million (ppm) relative to the NMR reference solvent signals (CDCl_3_: 7.26 ppm; *d*_6_-DMSO: 2.50 ppm). Multiplicities are indicated by s (singlet), br s (broad singlet), d (doublet), dd (doublet of doublet), t (triplet), dt (doublet of triplets), q (quartet) and m (multiplet). The coupling constants *J* are quoted in Hertz (Hz). LC-MS and HRMS-spectra were recorded using Waters Acquity UPLC®-system (with Acquity UPLC® BEH C18 column, 1.7 μm, 50 × 2.1 mm, Waters) with Waters Synapt G2 HDMS with the ESI (+), high resolution mode. The mobile phase consisted of H_2_O (A) and acetonitrile (B) both containing 0.1% HCOOH. Microwave syntheses were performed in sealed tubes using Biotage Initiator+ instrument equipped with an external IR sensor. The flash chromatography was performed with Biotage SP1 flash chromatography purification system with 254 nm UV-detector using SNAP KP-Sil 10, 25, 50 or 100 g cartridges. The TLC-plates were provided by Merck (Silica gel 60-F254) and visualization of the amine compounds was done using ninhydrin staining.

### 2-(3,5-Dibromo-4-hydroxyphenyl)ethan-1-amine hydrobromide (7)

Br_2_ (6.87 mL, 122 mmol, 2.5 equiv) was added dropwise (5 min) to a precooled solution of tyramine **6** (8.50 g, 49.0 mmol) in MeOH (40 mL) and the resulting mixture was stirred at 60°C for 14 h. The reaction mixture was then cooled to 0°C, filtered and the pale yellow solid residue was washed with Et_2_O (40 mL). The crude product was further dried under high vacuum to give **7** as a light yellow solid (15.3 g, 94%). ^1^H NMR (300 MHz, *d*_6_-DMSO) *δ* 9.77 (s, 1H), 7.77 (br s, 3H), 7.45 (s, 2H), 3.02 (t, *J* = 7.2 Hz, 2H), 2.77 (t, *J* = 7.4 Hz, 2H). ^13^C NMR (75 MHz, *d*_6_-DMSO) *δ* 149.9, 133.0, 132.1, 112.5, 40.1, 31.7. HRMS (TOF-ESI+): calcd for C_8_H_10_Br_2_NO [*M*+H]^+^: 293.9129, found: 293.9128.

### *tert*-Butyl (3,5-dibromo-4-hydroxyphenethyl)carbamate (8) [13]

A solution of di-*tert*-butyl dicarbonate (15.5 g, 71.1 mmol, 1.4 equiv) and triethylamine (17.7 mL, 127 mmol, 2.5 equiv) in MeOH (40 mL) was added dropwise to a stirred solution of **7** (16.8 g, 50.8 mmol) in MeOH (100 mL) under argon over a period of 15 min. The reaction mixture was then brought to rt, further stirred for 14 h and then concentrated *in vacuo*. EtOAc (30 mL) was added, washed with a 2 M solution of HCl in H_2_O (3 × 20 mL) and a saturated solution of NaHCO_3_ in water (3 × 20 mL). The organic layer was dried over anhydrous Na_2_SO_4_, filtered and evaporated to dryness to give crude **8** as a white solid (18.6 g, 93%). ^1^H NMR (300 MHz, CDCl_3_) *δ* 7.28 (s, 2H), 5.84 (s, 1H), 4.54 (br s, 1H), 3.31 (q, *J* = 6.7 Hz, 2H), 2.69 (t, *J* = 7.0 Hz, 2H), 1.44 (s, 9H).^13^C NMR (75 MHz, CDCl_3_) δ 155.9, 148.1, 133.8, 132.3, 110.0, 41.8, 34.9, 28.5. Note: one peak under CDCl_3._

### *tert*-Butyl [3,5-dibromo-4-[3-(dimethylamino)propoxy]phenethyl]carbamate (9) [4]

A mixture of **8** (18.6 g, 47.1 mmol), 3-chloro-*N*,*N*-dimethylpropan-1-amine hydrochloride (8.93 g, 56.5 mmol, 1.2 equiv), and K_2_CO_3_ (19.5 g, 141 mmol, 3.0 equiv) in acetone (150 mL) was refluxed under argon atmosphere for 24 h. It was then concentrated *in vacuo* and a 1 M solution of NaOH in H_2_O (100 mL) was added to the resulting residue, and the mixture was stirred for 10 min and extracted with dichloromethane (DCM, 100 mL). The organic layer was washed with a 1 M solution of NaOH in H_2_O (60 mL) and the combined aqueous phases were back-extracted with DCM (100 mL). The combined organic layers were dried over anhydrous Na_2_SO_4_ and concentrated to give a thick yellow liquid. This was then refluxed in *n*- hexane (180 mL) and allowed to crystallize to produce **9** as a white solid (18.1 g, 80%). Mp. 84.6–85.6 °C. ^1^H NMR (300 MHz, CDCl_3_) *δ* 7.31 (s, 2H), 4.58 (br s, 1H), 4.03 (t, *J* = 6.5 Hz, 2H), 3.31 (q, *J* = 6.8 Hz, 2H), 2.70 (t, *J* = 7.0 Hz, 2H), 2.59‒2.37 (m, 2H), 2.26 (s, 6H), 2.09–1.92 (m, 2H), 1.43 (s, 9H). ^13^C NMR (75 MHz, CDCl_3_) *δ* 155.9, 152.0, 137.7, 133.0, 118.4, 79.7, 72.1, 56.5, 45.7, 41.6, 35.2, 28.5, 28.5. HRMS (TOF-ESI+): calcd for C_18_H_29_Br_2_N_2_O_3_ [*M*+H]^+^: 479.0545, found: 479.0546.

### Purpurealidin E (4) [4]

A mixture of **9** (18.1 g, 37.6 mmol) and trifluoroacetic acid (TFA, 14.4 mL, 188 mmol, 5.0 equiv) in DCM (10 mL) was stirred under argon atmosphere for 24 h at room temperature. It was then concentrated by gentle air flow and the residual TFA was removed *in vacuo*. The residue was dissolved to EtOAc (100 mL) and washed with a 2 M solution of NaOH in H_2_O (2 × 50 mL). The aqueous phase was back-extracted with EtOAc (2 × 50 mL), the combined organic layers were dried over anhydrous Na_2_SO_4_ and concentrated to give **4** as a pale yellow liquid. ^1^H NMR spectrum showed some unreacted material, so the crude mixture was once again treated with TFA (20 mL + 10 mL) and stirred for another 36 h. The reaction mixture was then treated with a 10 M solution of NaOH in H_2_O (50 mL). H_2_O (25 mL) was added and extracted using EtOAc (3 × 100 mL). The combined organic layers were dried over anhydrous Na_2_SO_4_ and concentrated to give **4** as a pale yellow thick liquid (18 g, quant.). *R*_f_ 0.37 (DCM/MeOH, 4:1). ^1^H NMR (300 MHz, CDCl_3_) *δ* 7.33 (s, 2H), 4.03 (t, *J* = 6.5 Hz, 2H), 2.93 (t, *J* = 6.8 Hz, 2H), 2.64 (t, *J* = 6.7 Hz, 2H), 2.59–2.50 (m, 2H), 2.27 (s, 6H), 2.11–1.96 (m, 2H), 1.31 (br s, 2H). ^13^C NMR (75 MHz, CDCl_3_) *δ* 151.8, 138.7, 133.0, 118.3, 72.1, 56.5, 45.7, 43.3, 38.9, 28.5. HRMS (TOF-ESI+): calcd for C_13_H_21_Br_2_N_2_O [*M*+H]^+^: 379.0024, found: 379.0021.

### 3-Chloro-*N*-[3,5-dibromo-4-[3-(dimethylamino)propoxy]phenethyl]-4-methoxybenzamide (5)

A 20-mL MW tube was charged with 3-chloro-4-methoxybenzoic acid (0.59 g, 3.2 mmol, 1.5 equiv), 1-ethyl-3-(3-dimethylaminopropyl)carbodiimide hydrochloride (EDC·HCl, 0.76 g, 4.0 mmol, 1.5 equiv), and 1-hydroxybenzotriazole (HOBt, 0.53 g, 4.0 mmol, 1.5 equiv). The amine **4** (1.0 g, 0.26 mmol) was then added, followed by *N*,*N*-diisopropylethylamine (DIPEA, 0.69 mL, 4.0 mmol, 1.5 equiv). Dry DCM (15 mL) was added and the reaction mixture was irradiated with MW at 60°C for 2 h. The formation of the product was monitored with TLC (*n*-hexane with 1% Et_3_N-acetone 1:1). The reaction mixture was then diluted with DCM (10 mL) and washed with a 2 M solution of NaOH in H_2_O (2 × 20 mL). The combined aqueous layers were back-extracted with DCM (25 mL), dried over anhydrous Na_2_SO_4_ and evaporated to give the crude product (1.5 g). The crude products of two batches were combined (approx. 3.0 g) and subjected to flash chromatography (eluent: *n*-heptane, 1% Et_3_N: DCM, 1% Et_3_N, gradient 50–100%) to give **5** a white solid (1.0 g, 34%). This was repeated and the yield was found to be 30–35%. The batches were combined (6.12 g), subjected again to flash chromatography (eluent *n*-heptane, 2% Et_3_N: DCM, 2% Et_3_N, gradient 20–100%). The crude compound **5** was then treated with DCM and *n*-heptane (1:5) to make a flowing suspension of the product that was concentrated *in vacuo*. The resulting white solid was washed with *n*-heptane (5 × 10 mL) to give **5** as a white solid (5.84 g, 33% yield). ^1^H NMR (400 MHz, *d*_6_-DMSO) *δ* 8.49 (t, *J =* 5.5 Hz, 1H), 7.86 (d, *J =* 2 Hz, 1H), 7.79 (dd, *J =* 2.2, 8.7 Hz, 1H), 7.72 (s, 2H), 7.22 (d, *J =* 8.7 Hz, 1H), 3.94 (t, *J =* 6.5 Hz, 2H), 3.46 (t, 6.8 Hz, 2H), 2.80 (t, *J =* 6.9 Hz, 2H), 2.51 (t, *J =* 7.1 Hz, 2H); ^13^C NMR (101 MHz, CDCl_3_) *δ* 166.1, 157.6, 152.0, 137.5, 132.9, 129.0, 127.4, 126.9, 122.7, 118.4, 111.5, 77.4, 77.0, 76.7, 72.0, 56.4, 56.3, 45.5, 41.0, 34.5, 28.3. HRMS (ESI): calcd for C_21_H_26_N_2_O_3_ClBr_2_ [M+H] ^+^, 546.9999; found, 547.0002. More information about the synthesis of the analogs can be found in the Supporting Information (S1 Appendix).

### Expression of voltage-gated ion channels in *Xenopus laevis* oocytes

For the expression of hK_V_10.1a (GeneBank accession number: NM_002238.3) in *Xenopus* oocytes, the hK_V_10.1a-pSGEM plasmid was linearized with SfiI (ThermoFisher Scientific, Waltham, Massachusetts, USA) and transcribed using the T7 mMESSAGE-mMACHINE transcription kit (Ambion®, Carlsbad, California, USA).

Stage V-VI *Xenopus laevis* (African clawed frog) oocytes were isolated by partial ovariectomy. Mature female animals were purchased from Nasco (Fort Atkinson, Wisconsin, USA) and were housed in the Aquatic Facility (KU Leuven) in compliance with the regulations of the European Union (EU) concerning the welfare of laboratory animals as declared in Directive 2010/63/EU. The use of *Xenopus laevis* was approved by the Animal Ethics Committee of the KU Leuven (Project nr. P038/2017). Prior to harvesting the oocytes, the animals were anesthetized by a 15-min submersion in 0.1% tricaine methanesulfonate (pH 7.0). Isolated oocytes were defolliculated with 1.5 mg/mL collagenase.

Defolliculated oocytes were injected with 4 nL of cRNA at a concentration of 1 ng/nL using a micro-injector (Drummond Scientific®, Broomall, Pennsylvania, USA). The oocytes were incubated in a solution containing (in mM): NaCl, 96; KCl, 2; CaCl_2_, 1.8; MgCl_2_, 2 and HEPES, 5 (pH 7.4), supplemented with 50 mg/L gentamycin sulfate.

### Electrophysiological recordings

Two-electrode voltage-clamp recordings were performed at room temperature (18–22°C) using a Geneclamp 500 amplifier (Molecular Devices, USA) controlled by a pClamp data acquisition system (Axon Instruments®, Union City, California, USA). Whole cell currents from oocytes were recorded 1–4 days after injection. Bath solution composition was (in mM): NaCl, 96; KCl, 2; CaCl_2_, 1.8; MgCl_2_, 2 and HEPES, 5 (pH 7.5). Voltage and current electrodes were filled with a 3 M solution of KCl in H_2_O. Resistances of both electrodes were kept between 0.5 and 1.5 MΩ. The elicited K_V_10.1 currents were filtered at 1 kHz and sampled at 2 kHz using a four-pole low-pass Bessel filter. Leak subtraction was performed using a -P/4 protocol. K_V_10.1 currents were evoked by 2-s depolarizing pulses to 0 mV from a holding potential of -90 mV unless otherwise indicated.

### Live cell imaging

#### Cell cultures

Cell lines SH-SY5Y (ACC 209), DU145 (ACC 261), LNCaP (ACC 256) and NIH-3T3 (ACC 59) were purchased from DSMZ (Germany). MDA-MB-435S (HTB 129) and hTERT RPE-1 (CRL 4000) were obtained from ATCC (USA). Cell lines were cultured in their recommended media Supplemented with 10% or 15% FCS (PAA laboratories, Germany) at 37°C in humidified 5% CO_2_ atmosphere. Cell lines used in this study are given in Table 1. All media were purchased from ThermoFisher Scientific (Waltham, Massachusetts, United States).

**Table 1:** Cell lines used for proliferation, cytotoxicity and apoptosis assays.

Cell line	Description	Medium
SH-SY5Y	Human neuroblastoma cell line	RPMI + 15% FCS
DU145	Human prostate cancer cell line	DMEM + 10% FCS
LNCAP	Human prostate cancer cell line	RPMI + 15% FCS
NIH-3T3	Mouse embryonic fibroblast cell line	DMEM + 10% FCS
MDA-MB-435S	Human melanoma cell line	RPMI + 10% FCS
hTERT RPE-1	Human epithelial cell line	DMEM:F12 + 10% FCS + 10 μg/mL hygromycin B

#### Proliferation, cytotoxicity and apoptosis assays

Cell proliferation, cytotoxicity and apoptosis were assessed in a 96-well microtiter plate by live-cell imaging using an IncuCyte Zoom System (Essen BioScience, UK). Cell proliferation was monitored in terms of cell confluency (%). Cell cytotoxicity was assessed by the CellTox Green Dye assay (Promega, Madison, Wisconsin, USA). The Incucyte Caspase-3/7 apoptosis assay (Essen BioScience, UK) was used to evaluate the effect of compound **5** on the apoptotic pathway. As negative controls media supplemented with 0.05% of DMSO were used.

### Data analysis

All electrophysiological data are presented as means ± S.E.M of *n* ≥ 3 independent experiments unless otherwise indicated. All data was analyzed using pClamp Clampfit 10.4 (Molecular Devices®, Downingtown, Pennsylvania, USA) and OriginPro 8 (Originlab®, Northampton, Massachusetts, USA) or GraphPad Prism 5 software (GraphPad Software, Inc., San Diego, California, USA).

Live-cell imaging data were collected from the IncuCyte Zoom software and analyzed using GraphPad Prism 5 software. Proliferation was measured as Phase Object Confluence (%), cytotoxicity and apoptosis were measured as Green Object Count (1/mm^2^). All data are represented as mean S.E.M of *n* = 6 different wells.

## Results

### Compound synthesis

**Fig 2:**
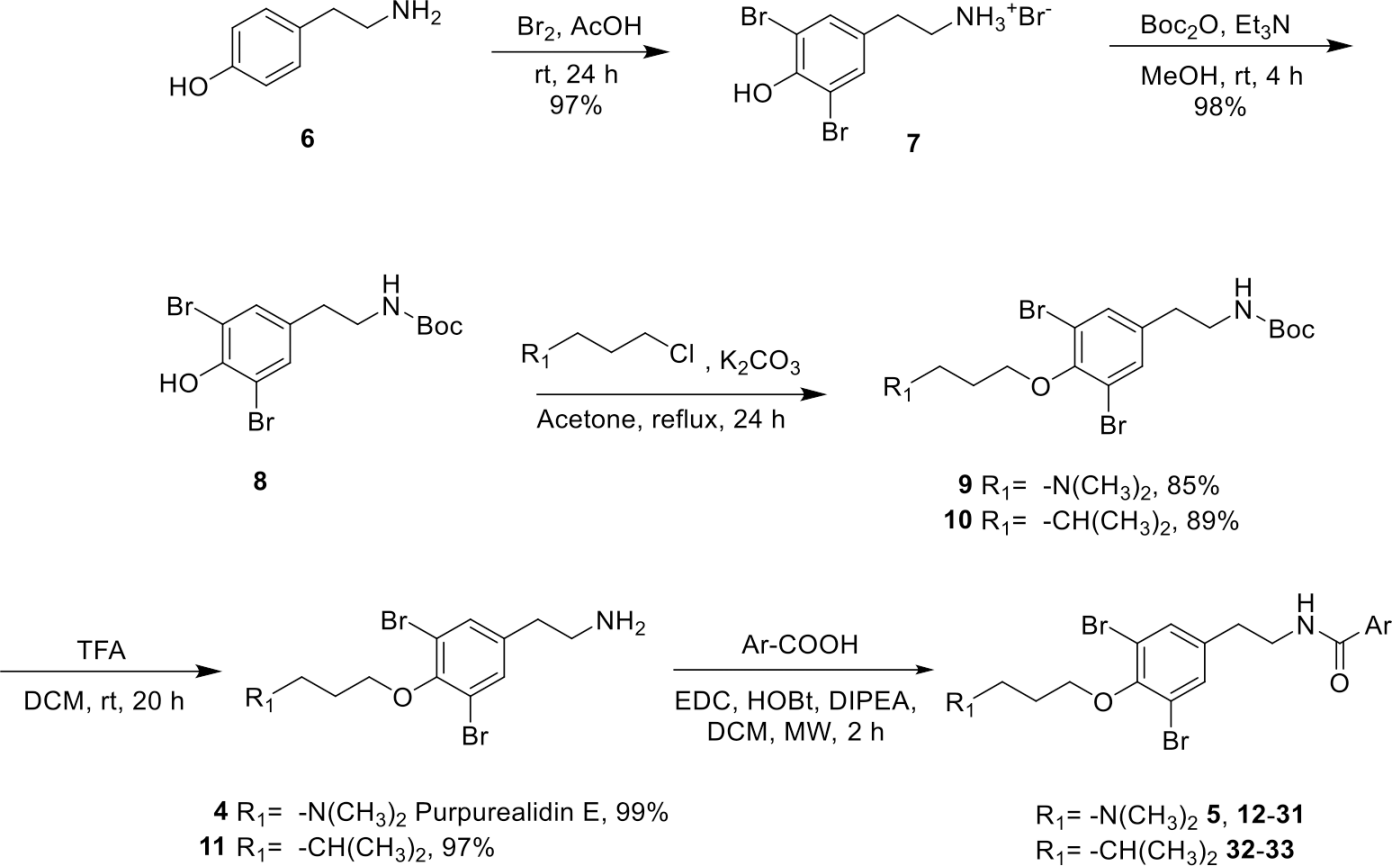
Synthesis of *N*,*N*-dimethyl amines (5, 12–31) and carbon analogs 32 and 33. The synthesis scheme of the *N*-monomethyl amine analogs 43–46 is depicted in Fig 3. The structures of the final compounds are presented in Fig 4.

Synthesis of the simplified purpurealidin analogs was based on the amide coupling of purpurealidin E **4** or tyramine derivative **11** with aromatic carboxylic acids. Purpurealidin E **4** and **11** were synthesized in four steps from tyramine in an overall yield of 80% and 82%, respectively, improving the literature yields (Fig 2) [4,13,14]. The synthetic route started with the bromination of tyramine. The dibrominated **7** was obtained in 97% yield followed by a straightforward *tert*-butyloxycarbonyl (Boc) protection of the amino moiety. The third step of the route was the alkylation of the phenolic hydroxyl using potassium carbonate as a base in acetone. The last step was a quantitative removal of Boc-protecting group to give compounds **4** and **11**. For the compounds **12**–**22**, **25**, **26**, **32** and **33** the amide coupling was carried out at room temperature using EDC-mediated coupling with the corresponding carboxylic acid in the presence of HOBt and DIPEA in DCM. The use of microwave irradiation at 60°C reduced the reaction time to 2 hours for compounds **5**, **23**, **24**, and **27**–**31**. A large scale synthesis of our hit compound **5** was achieved following the same synthetic sequence that was used for a small scale synthesis. This resulted in 5.8 g of the compound **5** in 23% overall yield.

Compound **34** with a free phenolic hydroxy group was synthesized to study the importance of the *N*,*N*-dimethylpropylamino chain for the activity. The alkylation led to the non-brominated analog **35**. This approach provides a short synthesis route and enables the variation of the amine moiety. Synthesis route for **35** is presented in the Supporting Information (S1 Appendix).

**Fig 3:**
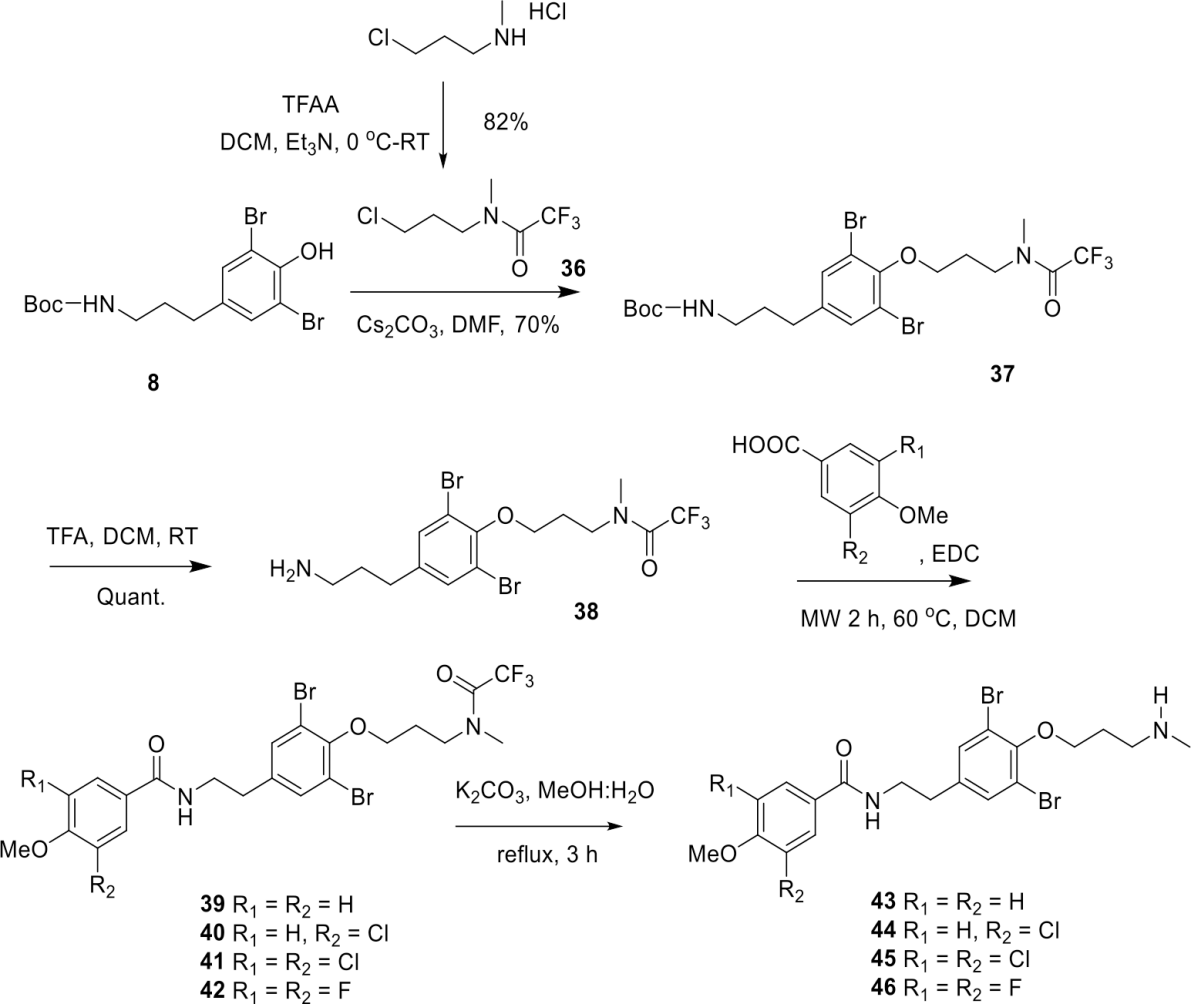
Synthesis of *N*-monomethyl amine analogs 43–46. The structures of the final compounds are presented in Fig 4.

*N*-monomethyl derivatives **43**–**46** were synthesized from the compound **8** using acid-stable trifluoroacetamide protecting group (Fig 3). Initially, the treatment of 3-chloromethyl amine hydrochloride with trifluoroacetic anhydride (TFAA) in the presence of triethylamine gave **36** in 82% yield. The subsequent *O*-alkylation of **8** with **36** in the presence of Cs_2_CO_3_ in *N*,*N*-dimethylformamide gave **37** in 70% yield. When **37** was treated with trifluoroacetic acid (TFA), the Boc group was selectively and quantitatively removed, whereas the trifluoroacetyl group remained intact. The resulting amine **38** was coupled with various aromatic carboxylic acids using EDC-mediated amide coupling under microwave conditions with 56–71% yields. Finally, removal of the trifluoroacetyl group using K_2_CO_3_ in MeOH gave *N*-monomethyl purpurealidin I analogs **43**–**46** (Fig 3).

### Synthetic purpurealidin analogs are able to inhibit K_V_10.1

**Fig 4:**
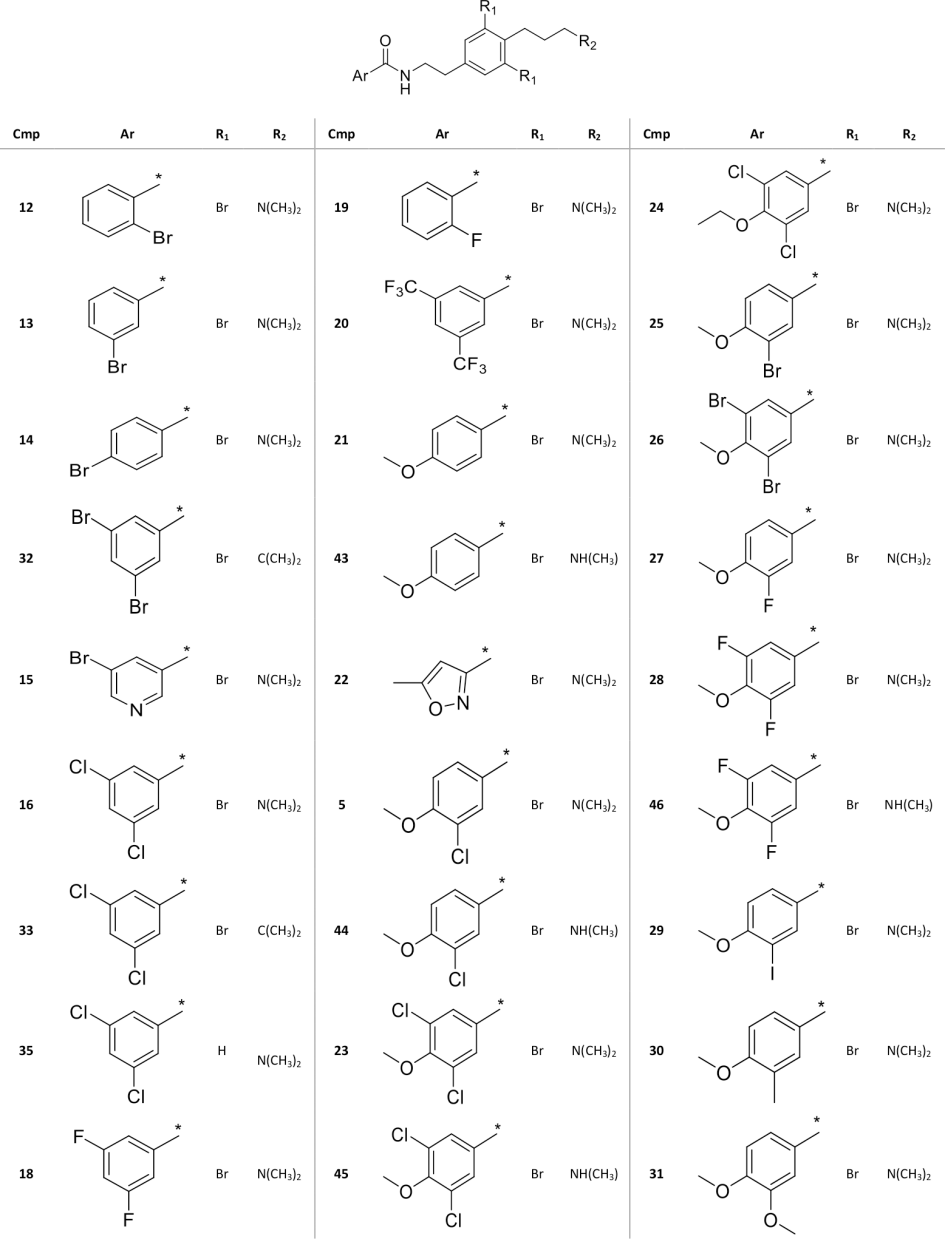
Structures of final amide compounds 5, 12–33, 35 and 43–46. Compound **34** is not shown in this overview, since it is considered as a precursor and not a final compound (see S1 Appendix).

**Fig 5:**
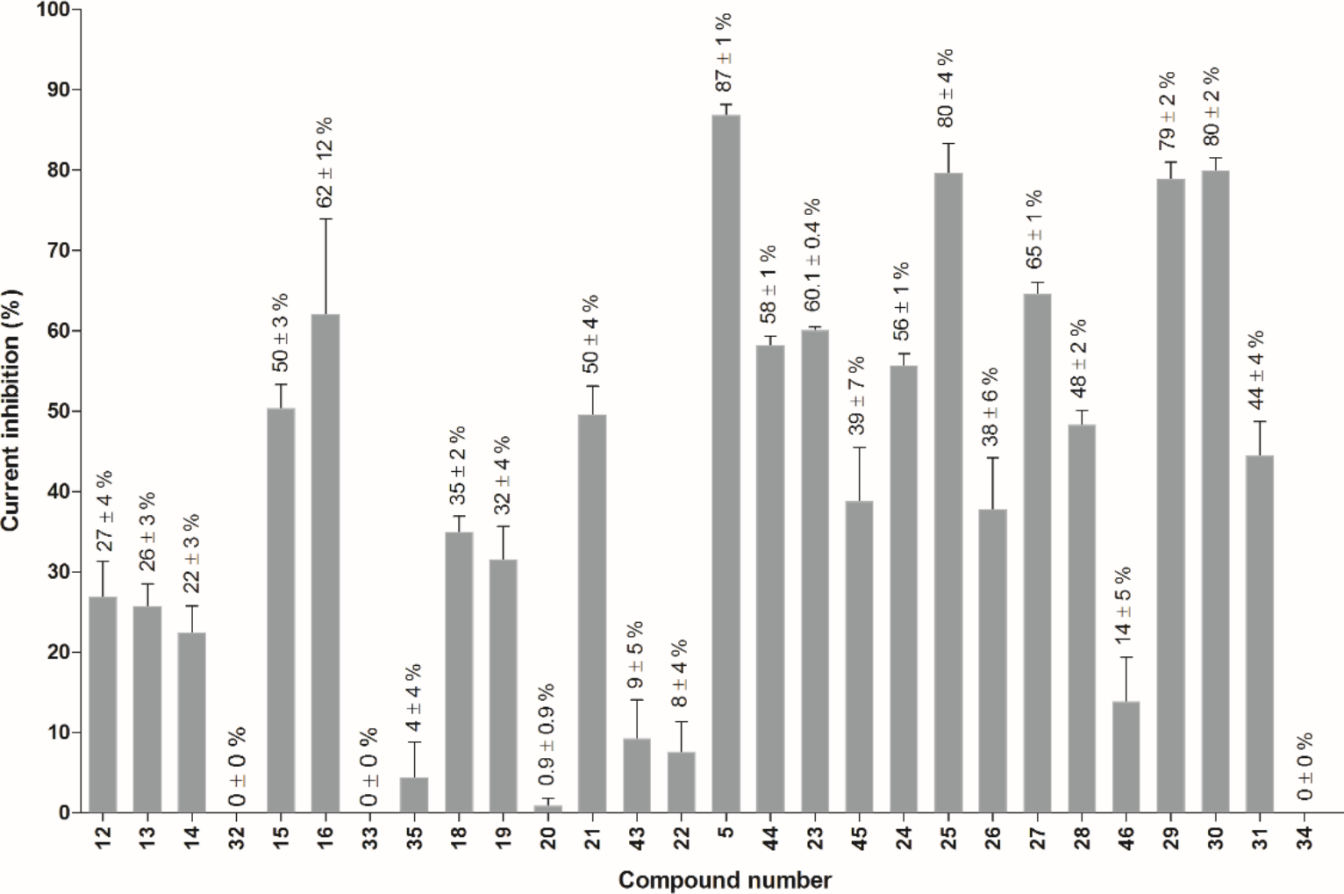
Overview of the K_V_10.1 current inhibition for each compound (40 μM). Compound numbers are assigned to the different analogs as shown in Fig 4. For each compound the K_V_10.1 current inhibition is given as average ± SEM.

Since secondary metabolites of marine sponges are known to possess a wide array of interesting bioactivities, their effect on the cancer-related potassium channel K_V_10.1 was evaluated. A preliminary screening showed that bromotyramine alkaloids were able to inhibit K_V_10.1 channels. In order to investigate the structure-activity relationship, 27 simplified bromotyramine analogs with the β-(hydroxyimino) amide parts replaced with amide moieties were synthesized and their effect on K_V_10.1 was electrophysiologically evaluated. In Fig 4 an overview of these 27 synthetic bromotyramine analogs, also known as the purpurealidin analogs is given. Their inhibitory effect on K_V_10.1 was evaluated by perfusion of 40 μM of each compound as an extracellular solution. The average current inhibition (%) is shown in Fig 5.

### Compound 5 inhibits K_V_10.1 in a dose- and voltage-dependent manner

**Fig 6:**
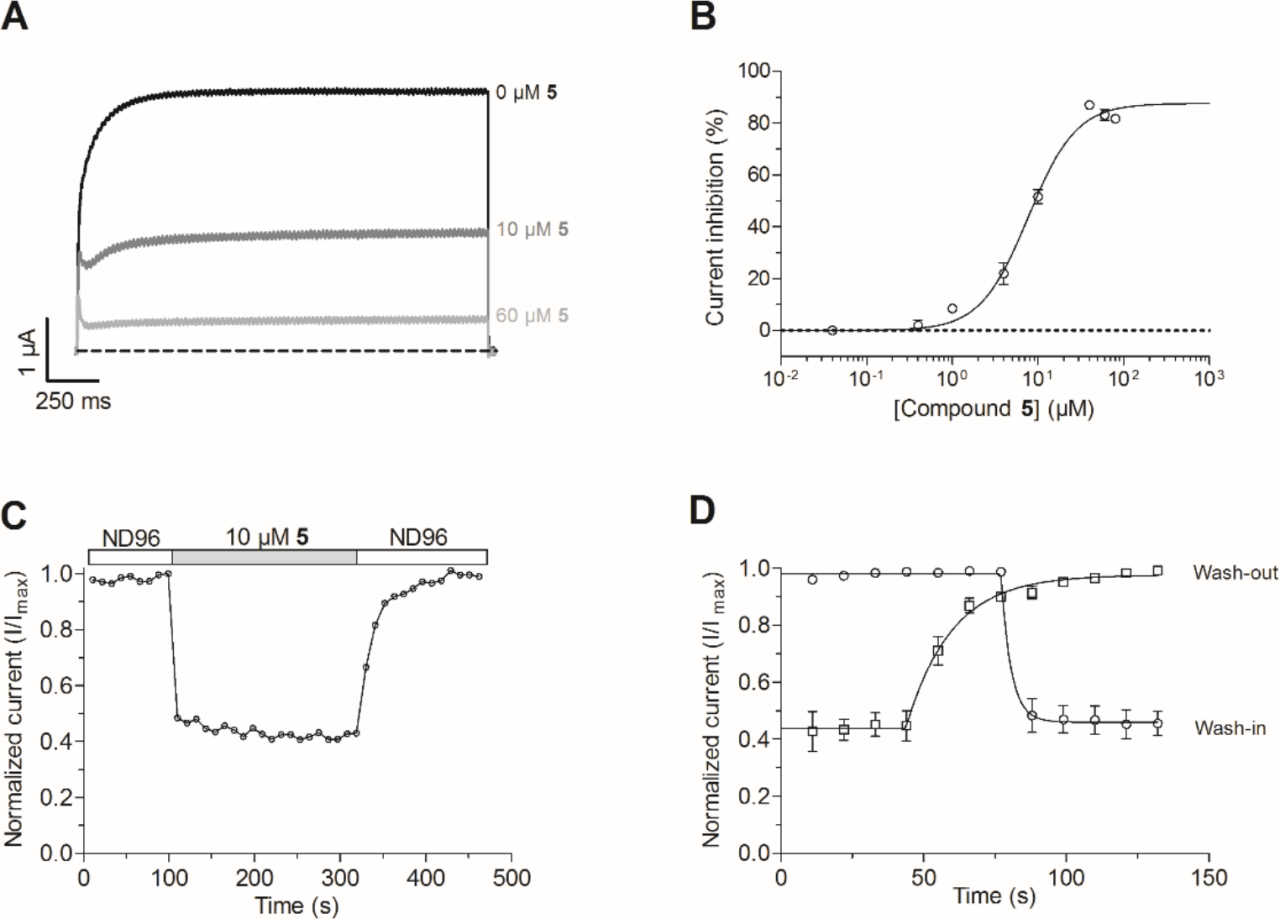
Compound 5 inhibits K_V_10.1 in a concentration-dependent and reversible manner. (A) A standard 2-s pulse protocol to 0 mV from the HP-90 mV was used. The K_V_10.1 current during ND96 (black), 10 μM **5** (dark grey) and 60 μM **5** (light grey) perfusion is shown. (B) Concentration-dependency of the induced K_V_10.1 inhibition by compound **5** is fitted with a logistic dose-response equation. (C) Normalized K_V_10.1 currents during wash-in and wash-out of compound **5** over time for one individual oocyte. (D) Averaged normalized K_V_10.1 currents during wash-in and wash-out of compound **5** over time. The time constants (τ) for wash-in and wash-out were respectively calculated with a one phase decay and an association equation.

To investigate the mechanism of action, the most potent purpurealidin derivative of this study, compound **5**, was selected. At 40 μM, compound **5** inhibits the K_V_10.1 current by 86.9 ± 1.3% (Fig 5). In Fig 6A, representative traces are shown in control situation (black line) and during perfusion of 10 μM (dark grey line) and 60 μM (light grey line) of **5**. At a concentration of 10 μM, compound **5** inhibits K_V_10.1 by 51 ± 3%, this inhibition is reversible as shown in Fig 6C. The concentration-dependency of the inhibition was further evaluated using 8 increasing concentrations ranging from 0.04 μM to 80 μM. To calculate the IC_50_ value, the curve was fitted with the logistic dose-response equation, y = $\frac{A_{1}\text{-}A_{2}}{1+{{(\mathrm{IC}}_{50}/[\mathrm{toxin}])}^{n_{H}}}$ + A_2_ where y represents the percentage of current inhibition, A_1_ the initial inhibition at the lowest toxin concentration (0%), A_2_ the final inhibition at the highest toxin concentration, IC_50_ the half maximal inhibitory toxin concentration and n_H_ the Hill coefficient. The calculated IC_50_-value and Hill coefficient are respectively 7.7 ± 1.0 μM and 1.6 ± 0.3 μM (Fig 6B). A more in-depth electrophysiological characterization was conducted with compound **5** at 10 μM, a concentration close to the IC_50_-value. In Fig 6C, a representative normalized time-dependent profile of the K_V_10.1 current during wash-in and wash-out of purpurealidin analog **5** is shown for one experiment. One sweep after perfusion with compound **5**, the expected inhibition of ± 50% is already reached. In Fig 6D the wash-in and wash -out time is estimated. Using a one phase decay equation y = ((y_0_ ‒ a)e^-t/τ^ + a) with y_0_ = 0.9795 and a = 0.43, the exponential time constant (τ) was determined as 5.3 s. The wash-out of **5** is slower than the wash-in (τ ~ 20s). This time constant was determined using a one phase association equation [y = y_0_ + (a ‒ y_0_)(1 ‒ e^-t/τ^)] with y_0_ = 0.4327 and a = 0.9858.

**Fig 7:**
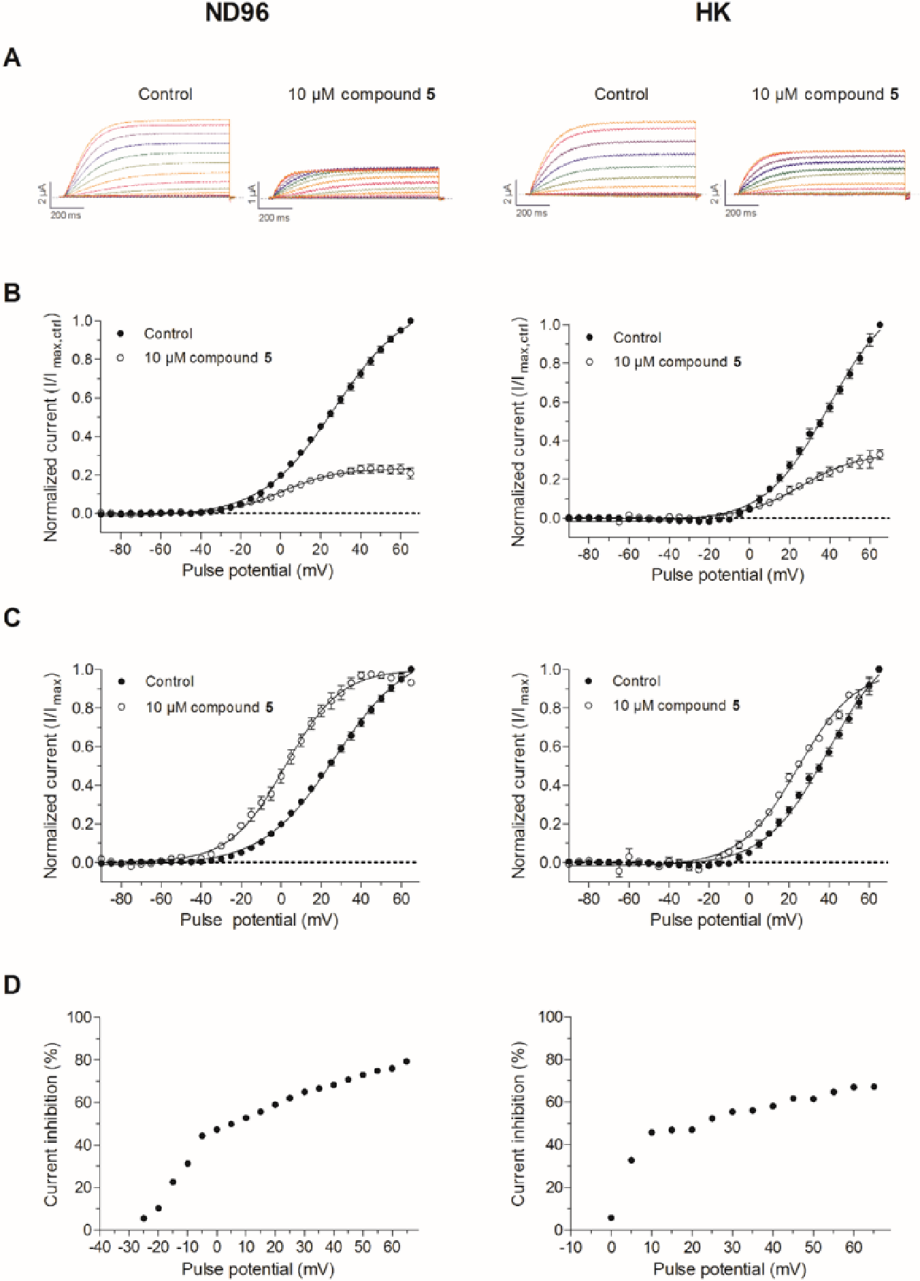
Evaluation of the voltage-dependency of the K_V_10.1 inhibition by compound 5. In the left panel, results obtained in a low extracellular potassium concentration (2 mM) are shown. In the right panel the same experiments were replicated in a high extracellular potassium concentration (96 mM). (A) Representative traces obtained in control condition and during perfusion of 10 μM **5** using an activation protocol as described in the text. (B) The normalized current (I/I_max,control_) in control condition (●) and during perfusion of 10 μM **5** (◯) are plotted against the pulse potential (mV). The curves are fitted with the Boltzmann equation. (C) The normalized current (I/I_max_) in control condition (●) and during perfusion of 10 μM **5** (◯) are plotted against the pulse potential (mV). The curves are fitted with the Boltzmann equation. (D) The current inhibition (%) at each pulse potential, calculated as (1-I_**5**_/I_ctrl_) × 100%, was plotted against its corresponding potential (mV).

To investigate the effect of compound **5** on the activation of K_V_10.1, 1-s activating steps from the holding potential -90 mV to 65 mV with 5 mV increments were applied. In Fig 7A (left) the representative traces are shown during perfusion of ND96 (control) and 10 μM of compound **5**. It appears that for pulse potentials ≥ 40 mV, the steady-state current amplitude reaches a plateau value in the presence of compound **5**. In the right panel, the experiments of the left panel were repeated with an external high potassium solution HK ([K^+^]_e_ = 96 mM). Here, this effect was less pronounced at the tested pulse potentials.

In Fig 7B, the normalized current amplitude (I/I_max,control_) in control condition (closed circles) and during **5** perfusion (open circles) is plotted against the pulse potential. The current-voltage curve reaches a plateau during **5** perfusion. This can indicate that apart from the modulation of the channel activation, compound **5** modulates K_V_10.1 in an additional manner.

In Fig 7C, the normalized current amplitude (I/I_max_) was plotted against the corresponding pulse potentials and fitted with the Boltzmann equation, y = $\frac{A_{1}\text{-}A_{2}}{1+e^{(\mathrm{V-}V_{1/2})/k}}$ + A_2_, where y represents the normalized current (I/I_max_), A_1_ is the initial y-value and A_2_ is the final y-value, I_max_ is the maximal current, V is the test voltage, V_1/2_ is the half-maximal voltage and k is the slope factor. A clear shift (ΔV_1/2_ = 21.0 ± 1.6 mV), from control condition (V_1/2_ = 23.2 ± 0.4 mV) to more negative potentials (V_1/2_ = 2.1 ± 0.4 mV) was observed. The slope factor (k_Comp5_ = 13.8 ± 0.3) was not significantly altered from control condition (k_control_ = 15.5 ± 0.4). A less pronounced shift was observed in HK solution (ΔV_1/2_ = 14.8 ± 2.1 mV), from control condition (V_1/2_ = 39.3 ± 1.7 mV) to more negative potentials (V_1/2_ = 24.5 ± 1.3 mV). The slope factor (k_Comp5_ = 14.5± 1.0) was not significantly altered from control condition (k_control_ = 15.7 ± 0.9).

When the current inhibition (%) is plotted against the applied pulse potential (Fig 7D), a clear voltage-dependent effect is observed. When the pulse potential increases, the current inhibition increases. This increase appears to be biphasic, an important increase until -5 mV (ND96) or 10 mV (HK) and a less pronounced increase at more depolarized potentials. At lower depolarizing potentials near the activating threshold, very little current inhibition is observed. However, when more channels open, the inhibitory effect initially increases fast.

**Fig 8:**
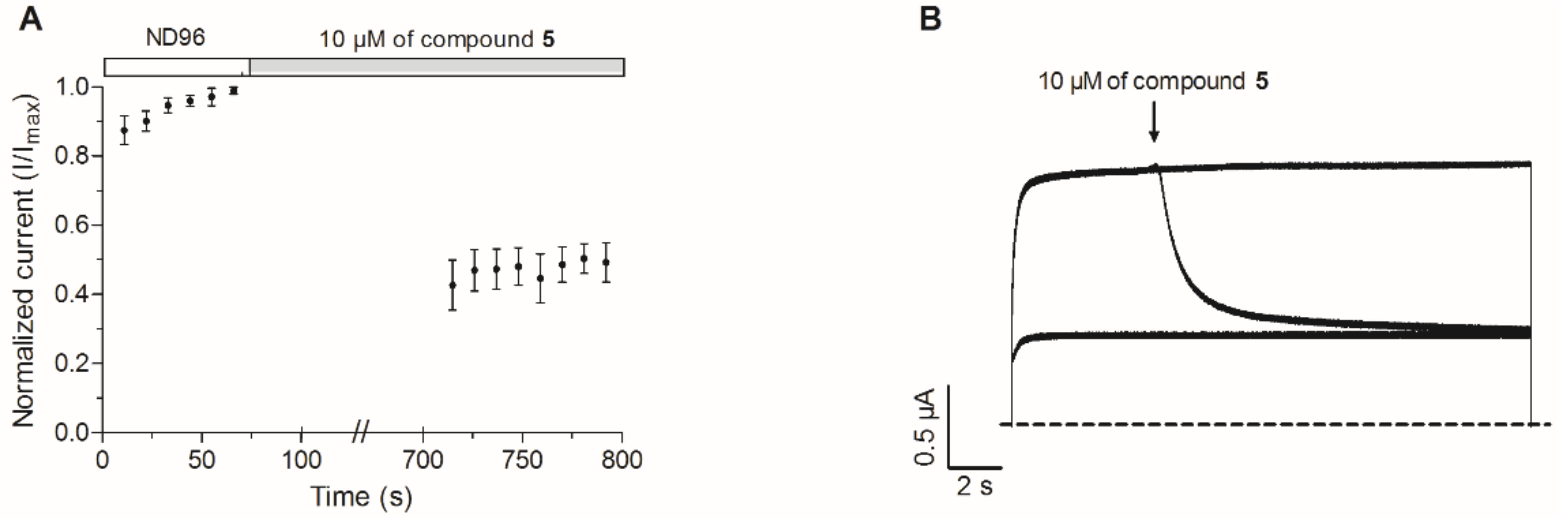
Evaluation of the state-dependency of the K_V_10.1 inhibition by compound 5. (A) During ND96 perfusion, a 2-s depolarizing pulse to 0 mV from the HP (-90 mV) was applied. The oocyte was then clamped at the HP and the extracellular solution was changed to 10 μM **5**. After 10 minutes, a 2-s depolarizing pulse to 0 mV was applied. (B) A 20-s depolarizing continuous step to 30 mV without P/4 leak subtraction was applied. During this step, the perfusion of ND96 was changed to a 10 μM of compound **5** (arrow).

To investigate if compound **5** binds specifically to open or closed K_V_10.1 channels, several electrophysiological protocols were used. In an initial experiment, K_V_10.1-expressing oocytes were perfused with 10 μM of compound **5**. The membrane potential of the oocytes was clamped at -90 mV prior to the addition of **5**. After 10 minutes, a 2-second depolarizing pulse to 0 mV was applied (Fig 8A). A 53 ± 4% current inhibition was observed, which corresponds indeed to the average current inhibition of K_V_10.1 by 10 μM of compound **5**. These data could suggest that purpurealidin analog **5** is able to bind to closed channels and is able to exert its effect through this binding. However, as was shown in Fig 6C and 6D, the inhibitory effect is very rapid which can skew this state-dependent observation. Therefore, an additional experiment was conducted, a 20-second depolarizing continuous step to 30 mV without P/4 leak subtraction was applied. During this step (around 5 s after the start of the activating step), the perfusion of ND96 was changed to 10 μM of compound **5**. After the addition of compound **5**, the current decreases (τ ~ 1.4 s) to approximately 50% of the control current (Fig 8B). This observation indicates that compound **5** is able to inhibit the potassium current through binding to open Kv10.1 channels.

### Compound 5 induces an apparent inactivation of K_V_10.1 channels

**Fig 9:**
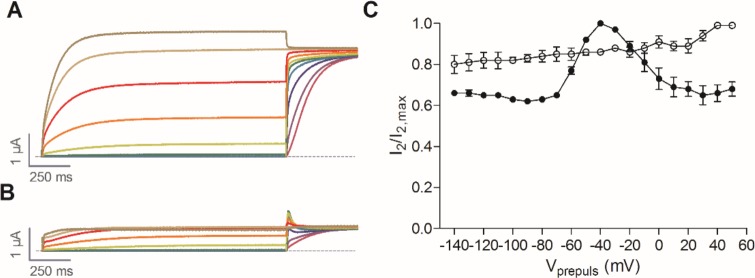
Voltage-dependence of K_V_10.1 inactivation induced by compound 5. (A) Representative trace of K_V_10.1 in control conditions during a two-pulse inactivation protocol. (B) Representative trace of K_V_10.1 during perfusion of 10 μM of compound **5** during a two-pulse inactivation protocol. (C) The non-inactivating channel fraction (I_2_/I_2,max_) in control situation (◯) and during perfusion of 10 μM of compound **5** (●) was plotted against the corresponding prepulse potential (mV).

This shift of the activation curve to more negative potentials, is reminiscent of the K_V_10.1 activation curve shift observed in the presence of mibefradil [15]. This Ca^2+^ channel antagonist was recently described as a gating modifier of K_V_10.1 by Gómez-Lagunas *et al*. Mibefradil induces an apparent inactivation from open (O) state and early closed states (C1). In order to evaluate if the shift of the activation curve to the left can be correlated with an induced inactivation, a two-pulse protocol was used as described in [15]. This protocol consists of a variable 1.5-s prepulse step, ranging from -140 mV to 50 mV in 10-mV steps, followed by a 0.5-s test pulse to 30 mV. In Fig 9 representative traces are shown in control condition (Fig 9A) and during compound **5** perfusion (Fig 9B). In control condition, the characteristic acceleration of activation with increasing prepulse potential was observed, but no apparent inactivation was detected. This is consistent with the literature since K_V_10.1 is presumed to be a non-inactivating or very slowly inactivating channel. However, during addition of **5**, an apparent inactivation is induced. Fig 9C shows the non-inactivating channel fraction (I_2_/I_2,max_) plotted against the corresponding prepulse potential. I_2_ is the peak current measured during the test pulse, I_2,max_ is the maximal peak current elicited during the consecutive test pulses. The non-inactivating channel fraction reaches a maximum around -50 mV, near the activation threshold of the K_V_10.1 channels. This means that when the channels start to open, the inactivated channel fraction reaches a minimum. This indicates that compound **5** induces an apparent open-state inactivation upon prolonged depolarizations and also affects the gating of the channel at hyperpolarized potentials.

### Competition experiment with mibefradil

**Fig 10:**
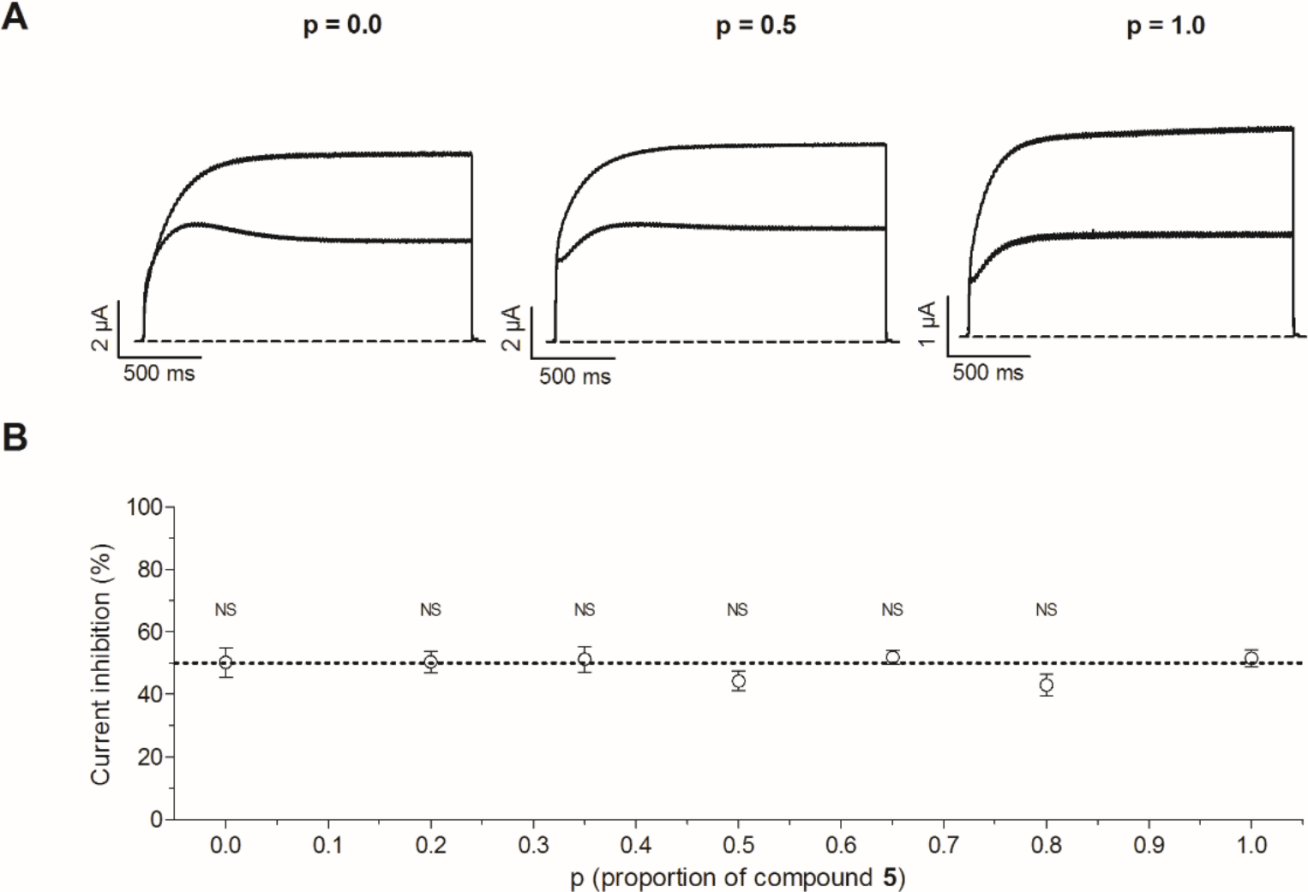
Competition plot with compound 5 and mibefradil. (A) Representative traces are shown in control condition and during perfusion of 10 μM mibefradil (p = 0.0), a mixture of 5 μM mibefradil and 5 μM of compound **5** (p = 0.5) and 10 μM of compound **5** (p = 1.0). (B) The current inhibition (%) observed after perfusion of the 7 different mixtures is plotted against p, which represents the fraction of compound **5** in the mixture.

Since the effect of compound **5** on K_V_10.1 is comparable to that of mibefradil (see discussion) [15], a competition experiment with both inhibitors was performed. This experiment was based on the competition plot of Chevillard and colleagues [16] and conducted as described by Gómez-Lagunas and colleagues [15]. This competition plot gives an indication whether two ligands compete for the same binding site on the target. First, the concentration of mibefradil that inhibits K_V_10.1 to the same extent as 10 μM of compound **5** (52 ± 3%) was determined. During 2-s depolarizing pulses to 0 mV, 10 μM of mibefradil inhibits K_V_10.1 by 50 ± 5%. These start concentrations will for now on be referred to as **5**_o_ and Mb_o_. Subsequently, the effect of several mixtures with different ratios/proportions (p) of compound **5** and mibefradil on K_V_10.1 was investigated. The solutions contained a mixture of p*5_o_ and (1-p)*Mb_o_ with p ranging from 0.0 to 1.0. For example; p = 0.0 means that the solution contains 0 μM **5** and 10 μM Mb, p = 0.2 indicates a solution of 2 μM **5** and 8 μM Mb *etc*. Representative traces for p = 0.0, p = 0.5 and p = 1.0 are shown in Fig 10A. The inhibitory effect of 7 different ratios was evaluated and the current inhibition (%) was plotted against the corresponding proportion (p) (Fig 10B). All data points appear to form a horizontal line, a two-tailed Student’s *t*-test was performed for each ratio against p = 1.0 (10 μM). This showed that there was no significant difference (NS). Since no clear maximum or minimum was reached, the competition plot indicates that the inhibitors compete for the same binding site.

### Compound 5 shows a cytotoxic and proapoptotic effect on a variety of cancerous and non-cancerous cell lines

To investigate if the K_V_10.1 modulator **5** is able to reduce the proliferation of K_V_10.1-overexpressing cell lines, the effect of different concentration of **5** was evaluated on a variety of mammalian cell lines. These cell lines included K_V_10.1-expressing cells such as the human neuroblastoma cell line SH-SY5Y, the human prostate cancer cell line DUI45, the human melanoma cell line MDA-MB-435S and the human epithelial cell line hTERT RPE-1. The effect of **5** was also investigated on two cell lines that are described to express an undetectable amount of K_V_10.1 channels, namely the human prostate cancer cell line LNCaP and the mouse embryonic fibroblast cell line NIH-3T3 (Table 2).

**Table 2:** Cell lines used for proliferation, cytotoxicity and apoptosis assays.

Cell line	Description	K_V_10.1 expression level	Ref.
SH-SY5Y	Human neuroblastoma cell line	High	[17]
DU145	Human prostate cancer cell line	Intermediate	[17]
LNCaP	Human prostate cancer cell line	Undetectable	[17]
NIH-3T3	Mouse embryonic fibroblast cell line	Undetectable	[3]
MDA-MB-435S	Human melanoma cell line	Moderate	[17]
hTERT RPE-1	Human epithelial cell line	Moderate	[17]

The following concentrations of compound **5** were added to the different cell lines, 100 μM, 50 μM, 20 μM, 10 μM, 5 μM and 2 μM to assess its effect on cell proliferation (S1 Fig) and cytotoxicity (S2 Fig). Indicative IC_50_-values for the proliferation assays were obtained by plotting the areas under the curve (AUC × 10^3^) against the tested compound **5** concentrations and by fitting the curve with a logistic dose-response equation. The EC_50_-values for the cytotoxicity assays were calculated correspondingly (S3 Fig). These calculated values are shown in Table 3. Although more data points would be necessary to calculate precise IC_50_ and EC_50_-values, it is clear that compound **5** inhibits the proliferation and induces cytotoxicity in all the tested cell lines in the low to middle micromolar range.

**Table 3:** Antiproliferative and cytotoxic effect of compound 5 on different cell lines.

Cell line	Antiproliferative effect (IC_50_)	Cytotoxic effect (EC_50_)
SH-SY5Y	12.22 μM	15.29 μM
DU145	28.87 μM	60.41 μM
LNCAP	26.42 μM	40.37 μM
NIH-3T3	7.13 μM	4.58 μM
MDA-MB-435S	28.06 μM	16.48 μM
hTERT RPE-1	12.48 μM	15.60 μM

A dose-dependent antiproliferative and cytotoxic effect was not only observed on the K_V_10.1-expressing cells but also on the control cell lines. It should be noted that a 48-h incubation of *Xenopus* oocytes with 10 μM concentration of compound **5** did not result in noticeable deterioration of the oocyte quality (visual and electrophysiological observation).

To evaluate the effect of compound **5** on the apoptotic pathway, 100 μM, 50 μM and 20 μM of **5** was added to the cell lines. After addition of the compound, caspase-3/7 activity was detected in all the cell lines, indicating an induction of the apoptotic pathway (S4 Fig).

## Discussion

Here we reported the identification of a novel group of K_V_10.1 inhibitors, namely the derivatives of the bromotyramine purpurealidin E from the marine sponge *Pseudoceratina purpurea*. In this study, mainly the carboxyl part of the tyramine amide was studied. Based on the K_V_10.1 inhibition results in Fig 5, the most active compound was 3-chloro-4-methoxyphenyl derivative **5**, and this substitution pattern also was confirmed in the case of 3-bromo-, 3-iodo- and 3-methyl-4-methoxyphenyl derivatives **25**, **29**, and **30**, respectively. These monohalogenated analogs were more active than dihalogenated 4-methoxy derivatives (**23**, **26**, **28**) or 3,4-dimethoxyphenol derivative **31**. Also 4-methoxyphenyl derivative **21** had moderate activity, so *para*-methoxy substitution seems to be important for K_V_10.1 inhibition. At the early stage of the study, 3,5-dichlorophenyl derivative showed promising inhibition and therefore it was used as a scaffold for structural modifications at the other end of the molecule. Replacement of *N*,*N*-dimethylamino moiety at the end of the propylamino chain by isopropyl (compounds **32** and **33**) caused a total loss of the activity and monomethylamine derivatives **43**–**46** were less active. Similarly, the lack of bromine atoms in the tyramine ring in compound **35** abolished the activity. Interestingly, no biological activity of purpurealidin E **4** has been previously reported to the best of our knowledge. Screening of secondary marine metabolites on a panel of ion channels could broaden are knowledge about the mode of action of these compounds and could result in the identification of novel ion channels ligands.

The most potent derivative, compound **5** exerts a concentration- and voltage-dependent inhibitory effect on Kv10.1 at low micromolar concentration. We suggest that compound **5** is a gating modifier that binds to the voltage sensor of K_V_10.1 near the binding site of mibefradil. Mibefradil is a Ca^2+^ channel antagonist and a K_V_10.1 gating modifier [15]. Like compound **5**, mibefradil shifts the activation curve to the left. Mibefradil seems to decrease the rate limiting step of K_V_10.1 activation and thereby facilitates the activation. Mibefradil also induces an apparent open-state inactivation upon prolonged depolarization (V_m_ ≥ -50 mV) and hyperpolarization (V_m_≤ -70 mV). At hyperpolarized potentials, channels dwell in early closed states (C1). It appears that at these potentials mibefradil induces a steady-state inactivation and stabilizes this inactivated state. At more depolarized potentials, when the channels are in an open state (O), mibefradil seems to induce an apparent open-state inactivation [15]. A similar effect is observed for compound **5**. Gómez-Lagunas *et al*. suggest that mibefradil binds to the S1-S4 voltage sensor module and alters the channel gating. Our competition plot data (Fig 10) indicates that the binding site of compound **5** on K_V_10.1 overlaps with the binding site of mibefradil. However, this hypothesis needs to be confirmed by site-directed mutagenesis studies. Both compounds are hydrophobic and carry one positive charge at pH 7.4 (S5 Fig). This suggests that they are both able to bind to hydrophobic and negatively charged residues of K_V_10.1.

Compound **5** shows a clear dose-dependent cytotoxic and proapoptotic effect on Kv10.1 expressing- and non-expressing cell lines. These observed effects can therefore not only be attributed to the effect of compound **5** on Kv10.1. Moreover, Kv10.1 is mostly described to be involved in cell proliferation [18,19] and migration [20]. An effect on apoptosis is not yet described. It is therefore presumed that the cytotoxic/proapoptotic effect of compound **5** is not or only in part due to its effect on K_V_10.1. To unravel the exact mode of action of this compound, more research is necessary.

Several sponge secondary metabolites, especially bromotyrosine derivatives, are known to induce cytotoxicity and/or apoptosis in mammalian cells [6]. For example, it has been proposed that the cytotoxic activity of psammaplin A, a natural bromotyrosine derivative from a marine sponge is due to the inhibition of several important enzymes (histone deacetylase, DNA methyltransferase *etc*.) by zinc chelation [21]. Psammaplin A also activates the peroxisome proliferator-activated receptor γ (PPARγ) and induces apoptosis in human breast cancer cells [22]. Zhang *et al*. suggested previously that K_V_10.1 is involved in the regulation of PPARγ expression [23].

## Conclusion

In this research paper, we investigated if simplified synthetic analogs of purpurealidins are able to inhibit the oncogenic potassium channel K_V_10.1 and if they exert antineoplastic effect on cancer cell lines. The purpurealidin E analog **5** shifts the K_V_10.1 activation curve to the left and induces an apparent inactivation. Since these effects are similar to those induced by the gating modifier mibefradil, a competition experiment was conducted. Our data suggests that analog **5** is a K_V_10.1 gating modifier that binds to voltage sensor domain of K_V_10.1 on the same binding site of mibefradil. Although compound **5** shows a cytototoxic effect on all the evaluated mammalian cell lines, it is still a valuable tool to study the gating of the cancer-related potassium channel K_V_10.1. Our study also shows that marine secondary metabolites are interesting compounds to consider in the search for novel ion channel ligands. These ligands cannot only be used as pharmacological tools to investigate disease-related ion channels but can also be used as templates for the design and synthesis of more potent and selective treatments for various channelopathies such as cancer, epilepsy, and diabetes.

## Supporting information

S1 Appendix Synthesis of purpurealidin analogs. (PDF)

S1 Fig Investigation of the antiproliferative effect of compound 5 on various cell lines. (PDF)

S2 Fig Investigation of the cytotoxic effect of compound 5 on various cell lines. (PDF)

S3 Fig Antiproliferative and cytotoxic effect of compound 5 on different cell lines. (PDF)

S4 Fig Investigation of the proapoptotic effect of compound 5 on various cell lines. (PDF)

S5 Fig pKa based protonation states of compound 5 and mibefradil. (PDF)
