# Supplementary material for: Synthesis of novel purpurealidin analogs and evaluation of their effect on the cancer-relevant potassium channel KV10.1
Source: PLoS One. 2017 Dec 8;12(12):e0188811. doi: 10.1371/journal.pone.0188811 (PMC5722316; doi:10.1371/journal.pone.0188811)
Supplement: S1 Appendix — (PDF) [file pone.0188811.s001.pdf]

S1 Appendix. Synthesis of purpurealidin analogs.

CONTENTS

**1. Chemistry experimental details**	**P2**
**2. ^{1}H and ^{13}C NMR spectra of compound 5**	**P16**
**3. References**	**P17**

1. Chemistry experimental details

General

All reactions were carried out using commercially available starting materials unless otherwise stated. The melting points were measured with Stuart SMP40 automated melting point apparatus and are uncorrected. ^{1}H NMR and ^{13}C NMR spectra in $CDCl_3$, d_6-DMSO, or CD_3OD at ambient temperature were recorded on a Varian Mercury *Plus* 300 spectrometer. Chemical shifts (δ) are given in parts per million (ppm) relative to the NMR reference solvent signals ($CDCl_3$: 7.26 ppm; CD_3OD: 3.31 ppm; d_6-DMSO: 2.50 ppm). Multiplicities are indicated by s (singlet), br s (broad singlet), d (doublet), dd (doublet of doublet), t (triplet), dt (doublet of triplets), q (quartet), m (multiplet) and m_c (centralized multiplet).The coupling constants J are quoted in Hertz (Hz). LC-MS and HRMS-spectra were recorded using Waters Acquity UPLC®-system (with Acquity UPLC® BEH C18 column, 1.7 µm, 50 × 2.1 mm, Waters) with Waters Synapt G2 HDMS with the ESI (+), high resolution mode. The mobile phase consisted of H_2O (A) and acetonitrile (B) both containing 0.1% HCOOH. Microwave syntheses were performed in sealed tubes using Biotage Initiator+ instrument equipped with an external IR sensor. The flash chromatography was performed with Biotage SP1 flash chromatography purification system with 254 nm UV-detector using SNAP KP-Sil 10, 25, 50 or 100 g cartridges. The TLC-plates were provided by Merck (Silica gel 60-F254) and visualization of the amine compounds was done using ninhydrin staining.

Small scale syntheses

2-(3,5-Dibromo-4-hydroxyphenyl)ethan-1-amine hydrobromide (7)

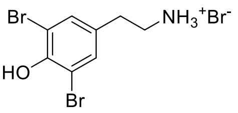

Tyramine **6** (1.00 g, 7.29 mmol) was dissolved in AcOH (15 mL). After 15 min, bromine (1.31 mL, 25.5 mmol, 3.5 equiv) was added dropwise over a period of 10 min. The reaction mixture was stirred for 26 h at room temperature. The mixture was quenched with Et_2O (15 mL). The product was filtered and washed with Et_2O (40 mL) to give a white solid (2.67 g, 97% yield). For the spectral data, please refer to the main text of the article.

tert-Butyl (3,5-dibromo-4-hydroxyphenethyl)carbamate (8) [1]

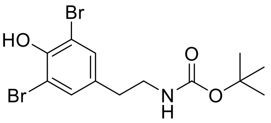

2-(3,5-Dibromo-4-hydroxyphenyl)ethan-1-amine hydrobromide (**6**, 2.62 g, 6.97 mmol) and Boc_2O (1.82 g, 8.36 mmol, 1.2 equiv) were dissolved in anhydrous MeOH (50 mL). TEA (2.91 mL, 20.9 mmol, 3.0 equiv) was added dropwise over a period of 4 min. The reaction mixture was stirred at room temperature. TLC (*n*-hexane/EtOAc, 1:1) indicated that the reaction was completed after 4 h. The solvent was removed *in vacuo* and the crude product was extracted with EtOAc (40 mL) and washed with a 0.5 M solution of HCl in H_2O (40 mL). The aqueous phase was re-extracted with EtOAc (2 × 25 mL). The combined organic phases were washed with water three times until pH was neutral (2 × 20 mL, 1 × 40 mL), dried over anhydrous Na_2SO_4, filtered and the solvent was evaporated *in vacuo*. A white solid (2.71 g, 98% yield).

tert-Butyl [3,5-dibromo-4-[3-(dimethylamino)propoxy]phenethyl]carbamate (9) [2]

tert-Butyl (3,5-dibromo-4-hydroxyphenethyl)carbamate (**8**, 2.0 g, 5.06 mmol), 3-chloro-*N*,*N*-dimethylpropan-1-amine hydrochloride (0.96 g, 6.07 mmol, 1.2 equiv) and anhydrous K_2CO_3 (2.10 g, 15.2 mmol, 3.0 equiv) were suspended in dry acetone (30 mL) under argon atmosphere. The mixture was refluxed for 24 h. The solvent was removed *in vacuo*, a 0.1 M solution of NaOH in H_2O (40 mL) was added and the resulting mixture was extracted with EtOAc (2 × 40 mL). The combined organic layers were washed with water (2 × 20 mL). The organic phase was dried with anhydrous Na_2SO_4, filtered and the solvent was removed *in vacuo* to give a white solid. Recrystallization from *n*-hexane yielded a white crystalline solid (2.21 g, 92%). For the spectral data, please refer to the main text of the article.

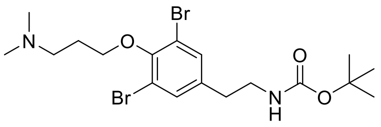

tert-Butyl [3,5-dibromo-4-[(4-methylpentyl)oxy]phenethyl]carbamate (10)

Compound **8** (0.50 g, 2.27 mmol) and anhydrous K_2CO_3 (0.44 g, 3.16 mmol, 2.5 equiv) were suspended in anhydrous acetone (3 mL) under argon atmosphere and 1-bromo-4-methylpentane (0.23 mL, 1.58 mmol, 1.25 equiv) was added. The resulting mixture was refluxed for 23 h. The solvent was then removed *in vacuo*. H_2O (20 mL) was added and the resulting mixture was extracted with dichloromethane (DCM, 2 × 20 mL). The combined organic layers were dried with anhydrous Na_2SO_4, filtered and the solvent was removed *in vacuo*. The product was recrystallized from *n*-hexane to give white crystals (0.54 g, 89% yield). Mp. 108–110 °C. ^{1}H NMR (300 MHz, $CDCl_3$) δ 7.32 (s, 2H), 4.53 (br s, 1H), 3.97 (t, J = 6.7 Hz, 2H), 3.32 (q, J = 6.8 Hz, 2H), 2.71 (t, J = 7.0 Hz, 2H), 1.95–1.72 (m, 2H), 1.72–1.55 (m, 1H), 1.44 (s, 9H), 1.43–1.36 (m, 2H), 0.93 (d, J = 6.6 Hz, 6H). ^{13}C NMR (75 MHz, $CDCl_3$) δ 155. 9, 152.2, 137.6, 133.0, 118.5, 74.0, 41.6, 35.1, 28.6, 28.1, 28.1, 22.7. HRMS (TOF-ESI+): calcd for $C_{19}H_{29}Br_2NO_3Na$ $[M+Na]^+$: 500.0412, found: 500.0416.

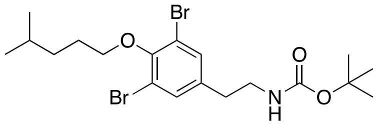

General method for Boc-cleavage of 4 and 9

tert-Butylcarbamate **9** or **10** was dissolved in anhydrous DCM (10-16 mL) under argon atmosphere. Trifluoroacetic acid (TFA, 5-8 mL, excess) was added dropwise. The resulting mixture was stirred under argon atmosphere at room temperature for 20 h. The reaction mixture was then concentrated using air flow. The remaining solvent and TFA were removed *in vacuo*. The crude mixture was dissolved to EtOAc and washed with a 2 M solution of NaOH in H_2O. The aqueous phase was re-extracted with EtOAc and the combined organic layers were washed with water, dried over anhydrous Na_2SO_4, filtered and the solvent was removed *in vacuo.*

Purpurealidin E (4) [2]

A light yellow oil (0.86 g, 99% yield). For the spectral data, please refer to the main text of the article.

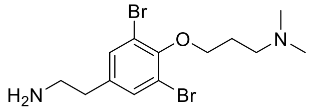

2-[3,5-Dibromo-4-[(4-methylpentyl)oxy]phenyl]ethan-1-amine (11)

A yellow oil (0.39 g, 97% yield). ^{1}H NMR (300 MHz, $CDCl_3$) δ 7.33 (s, 2H), 3.96 (s, 2H), 2.94 (t, J = 6.8 Hz, 2H), 2.70–2.59 (m, 2H), 1.94–1.78 (m, 2H), 1.74–1.54 (m, 1H), 1.48–1.36 (m, 2H), 1.32 (br s, 2H), 0.93 (d, J = 6.6 Hz, 6H). ^{13}C NMR (75 MHz, $CDCl_3$) δ 152.0, 138.5, 133.0, 118.4, 74.0, 43.3, 38.8, 35.1, 28.1, 28.0, 22.7.

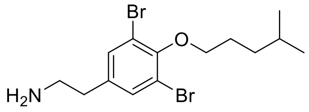

A general procedure: Synthesis of amides

The following compounds were synthesized using general procedures A or B:

Method A

Purpurealidin E **4** or its carbon analog **11** (0.26–0.30 mmol), the corresponding carboxylic acid (1.2 equiv), 1-ethyl-3-(3-dimethylaminopropyl)carbodiimide (EDC) hydrochloride (1.2 equiv), and 1-hydroxybenzotriazole (HOBt, 1.2 equiv) were dissolved in anhydrous DCM (7 mL) under argon atmosphere. *N,N*-Diisopropylethylamine (DIPEA, 3.0 equiv) was added dropwise to the mixture, and the resulting mixture was then stirred at room temperature under argon atmosphere for 2–4 d. The reaction mixture was quenched with H_2O (4 mL). The resulting mixture was washed with a 1 M solution of NaOH in H_2O (10 mL) and extracted with DCM (10 mL). The aqueous phase was re-extracted with DCM (2 × 10 mL). The combined organic layers were washed with H_2O (40 mL). The aqueous phase was re-extracted with DCM (2 × 20 mL). The combined organic layers were dried over anhydrous Na_2SO_4, filtered and the solvent was removed *in vacuo*. The crude product was purified by flash chromatography (DCM/MeOH gradient 0→20% or *n*-hexane/EtOAc gradient: 0→100%).

2-Bromo-*N*-[3,5-dibromo-4-[3-(dimethylamino)propoxy]phenethyl]benzamide (12)

A light yellow oil (91.0 mg, 46% yield). R_f 0.36 (*n*-hexane/acetone, 50% + 3% Et_3N). ^{1}H NMR(300 MHz, $CDCl_3$) δ 7.56 (dd, J = 1.3, 7.9 Hz, 1H), 7.48 (dd, J = 1.8, 7.6 Hz, 1H,), 7.41 (s, 2H), 7.34 (ddd, J = 1.3, 7.5, 7.6 Hz, 1H), 7.25 (ddd, J = 1.8, 7.5, 7.9 Hz, 1H), 6.09 (br t, J = 5.3 Hz, 1H), 4.03 (t, J = 6.5 Hz, 2H), 3.72–3.63 (m, 2H), 2.88 (t, J = 6.9 Hz, 2H), 2.54 (t, J = 7.4, 2H), 2.26 (s, 6H), 2.10–1.96 (m, 2H); ^{13}C NMR (75 MHz, $CDCl_3$) δ 167.8, 152.2, 137.8, 137.4, 133.5, 133.2, 131.5, 129.7, 127.7, 119.3, 118.5, 72.2, 56.5, 45.7, 41.1, 34.3, 28.5; HRMS (TOF-ESI+): calcd for $C_{20}H_{24}Br_3N_2O_2$ $[M+H]^+$: 560.9388, found: 560.9388.

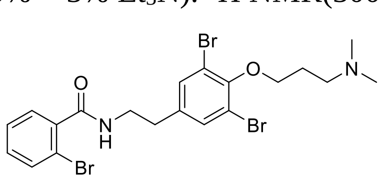

3-[2,6-Dibromo-4-[2-(3-bromobenzamido)ethyl]phenoxy]-*N,N*-dimethylpropan-1-aminium hydrochloride (13)

A free base as a yellow oil (73.0 mg). ^{1}H NMR (300 MHz, $CDCl_3$) δ 7.86 (t, J = 1.8 Hz, 1H), 7.63 (t, J = 1.9 Hz, 1H), 7.61 (t, J = 1.9 Hz, 1H), 7.37 (s, 2H), 7.30 (t, J = 7.8 Hz, 1H), 4.05 (t, J = 6.4 Hz, 2H), 3.65 (q, J = 6.8 Hz, 2H), 2.89–2.82 (m, 2H), 2.61–2.52 (m, 2H), 2.29 (s, 6H), 2.06 (dt, J = 6.5, 13.5 Hz, 2H).

The oil was dissolved in 1,4-dioxane (2 mL) and to the mixture was added a 4 M solution of HCl in 1,4-dioxane (250 µL). The resulting salt was filtered and washed with Et_2O to give a light yellow solid (59.0 mg, 76% yield). ^1H NMR (300 MHz, CD_3OD) δ 7.97 (t, J = 1.7 Hz, 1H), 7.86–7.69 (m, 2H), 7.57 (s, 2H), 7.44 (t, J = 7.9 Hz, 1H), 4.18 (t, J = 5.4 Hz, 2H), 3.68–3.52 (m, 4H), 3.03 (s, 6H), 2.93 (t, J = 7.0 Hz, 2H), 2.37 (dt, J = 5.3, 9.3 Hz, 2H); ^{13}C NMR (75 MHz, CD_3OD) δ 168.61, 152.19, 140.45, 137.82, 135.53, 134.51, 131.44, 131.32, 126.98, 123.49, 118.77, 71.17, 57.12, 43.76, 42.01, 35.10, 26.38; HRMS (TOF-ESI+): calcd for $C_{20}H_{25}Br_3N_2O_2$ $[M+H]^+$: 560.9388, found: 560.9388.

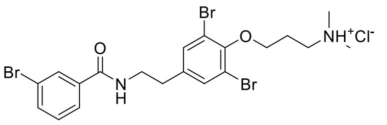

4-Bromo-*N*-[3,5-dibromo-4-[3-(dimethylamino)propoxy]phenethyl]benzamide (14)

A white solid (98.0 mg, 59% yield). R_f 0.31 (*n*-hexane/acetone, 3:2 + 3% Et_3N). ^1H NMR (300 MHz, $CDCl_3$) δ 7.63–7.52 (m, 4H), 7.36 (s, 2H), 6.28 (br t, J = 5.9 Hz, 1H), 4.05 (t, J = 6.2 Hz, 2H), 3.64 (q, J = 6.8 Hz, 2H), 2.88–2.74 (m, 4H), 2.43 (s, 6H), 2.22–2.07 (m, 2H); ^{13}C NMR (75 MHz, $CDCl_3$) δ 166.6, 151.7, 137.6, 133.2, 132.9, 131.8, 128.5, 126.3, 118.3, 71.3, 56.2, 44.8, 41.0, 34.4, 27.4; HRMS (TOF-ESI+): calcd for $C_{20}H_{24}Br_3N_2O_2$ $[M+H]^+$: 560.9388, found: 560.9387.

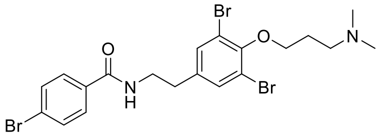

3,5-Dibromo-*N*-[3,5-dibromo-4-[(4-methylpentyl)oxy]phenethyl]benzamide (32)

A white solid (0.100 g, 59% yield). Mp. 123-124 °C. R_f 0.78 (*n*-hexane/EtOAc, 1:1). ^1H NMR (300 MHz, $CDCl_3$) δ 7.79–7.77 (m, 1H), 7.76–7.74 (m, 2H), 7.36 (s, 2H), 6.16 (t, J = 5.9 Hz, 1H), 3.98 (t, J = 6.7 Hz, 2H), 3.69–3.53 (m, 2H), 2.84 (t, J = 7.0 Hz, 2H), 1.97–1.79 (m, 2H), 1.72–1.51 (m, 1H), 1.48–1.31 (m, 2H), 0.93 (d, J = 6.6 Hz, 6H); ^{13}C NMR (75 MHz, $CDCl_3$) δ 165.0, 152.5, 137.9, 137.1, 133.0, 129.0, 123.5, 118.7, 74.1, 41.3, 35.1, 34.5, 28.1, 28.0, 22.7; HRMS (TOF-ESI+): calcd for $C_{21}H_{24}Br_4NO_2$ $[M+H]^+$: 637.8541, found: 637.8537.

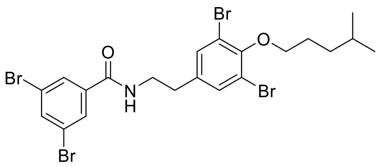

5-Bromo-*N*-[3,5-dibromo-4-[3-(dimethylamino)propoxy]phenethyl]nicotinamide (15)

A white solid (84.0 mg, 57% yield). R_f 0.29 (*n*-hexane/acetone, 3:2 + 3% Et_3N). ^1H NMR (300 MHz, $CDCl_3$) δ 8.80 (d, J = 1.9 Hz, 1H), 8.77 (d, J = 2.3 Hz, 1H), 8.21 (t, J = 2.1 Hz, 1H), 7.36 (s, 2H), 6.43 (br t, J = 5.5 Hz, 1H), 4.04 (t, J = 6.4 Hz, 2H), 3.71–3.62 (m, 2H), 2.86 (t, J = 7.0 Hz, 2H), 2.60 (t, J = 7.4 Hz, 2H), 2.31 (s, 6H), 2.13–1.97 (m, 2H); ^{13}C NMR (75 MHz, $CDCl_3$) δ 164.5, 153.6, 152.2, 145.9, 137.9, 137.3, 133.0, 131.6, 121.2, 118.6, 72.0, 56.5, 45.5, 41.3, 34.5, 28.2; HRMS (TOF-ESI+): calcd for $C_{19}H_{23}Br_3N_3O_2$ $[M+H]^+$: 561.9340, found: 561.9339.

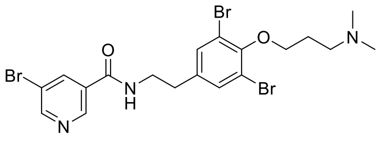

3,5-Dichloro-*N*-[3,5-dibromo-4-[3-(dimethylamino)propoxy]phenethyl]benzamide (16).

Recrystallization from *n*-hexane and DCM gave a white solid (19.0 mg, 13%). Mp. 83-84.5 °C. R_f 0.42 (1:1 DCM:MeOH + Et_3N 1%). ^{1}H NMR (300 MHz, $CDCl_3$) δ 7.57 (d, J = 1.9 Hz, 2H), 7.49 (t, J = 1.9 Hz, 1H), 7.37 (s, 2H), 6.09 (s, 1H), 4.06 (t, J = 6.4 Hz, 2H), 3.65 (q, J = 6.7 Hz, 2H), 2.85 (t, J = 7.0 Hz, 2H), 2.63 (t, J = 7.7 Hz, 2H), 2.33 (s, 6H), 2.09 (m, 2H); ^{13}C NMR (75 MHz, $CDCl_3$) δ 165.22, 152.28, 137.48, 137.32, 135.77, 133.06, 131.64, 125.70, 118.65, 72.00, 56.48, 45.45, 41.30, 34.52, 28.19; FT-IR: 3305 (w), 2941, 2866, 2829, 2781, 1647, 1562, 1546, 1452, 1250, 1050, 808, 744; HRMS (TOF-ESI+): calcd for $C_{20}H_{23}N_2O_2Cl_2Br_2$ $[M+H]^+$: 550.9503, found: 550.9503.

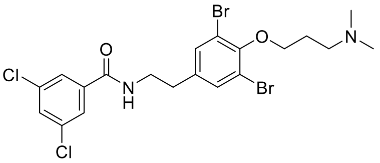

3,5-Dichloro-*N*-[3,5-dibromo-4-[(4-methylpentyl)oxy]phenethyl]benzamide (33)

A white solid (89.0 mg, 63% yield). Mp. 107.5-109 °C. 1H NMR (300 MHz, $CDCl_3$) δ 7.56 (d, J = 1.9 Hz, 2H), 7.48 (t, J = 1.9 Hz, 1H), 7.36 (s, 2H), 6.16 (br t, J = 3.9 Hz, 1H), 3.98 (t, J = 6.7 Hz, 2H), 3.64 (q, J = 6.9 Hz, 2H), 2.84 (t, J = 7.0 Hz, 2H), 1.95–1.77 (m, 2H), 1.71–1.54 (m, 1H), 1.50–1.32 (m, 2H), 0.93 (d, J = 6.6 Hz, 6H); ^{13}C NMR(75 MHz, $CDCl_3$) δ 165.3, 152.5, 137.5, 137.1, 135.7, 133.0, 131.6, 125.7, 118.7, 74.1, 41.3, 35.1, 34.5, 28.1, 28.0, 22.7; HRMS (TOF-ESI+): calcd for $C_{21}H_{24}Br_2Cl_2NO_2$ $[M+H]^+$: 549.9551, found: 549.9554.

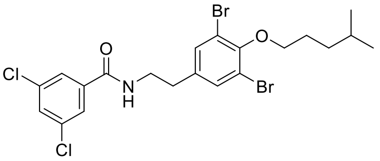

***N*-[3,5-Dibromo-4-[3-(dimethylamino)propoxy]phenethyl]-3,5-difluorobenzamide (18)**

A white solid (30.0 mg, 21% yield). R_f 0.33 (*n*-hexane/acetone, 3:2 + 3% Et_3N). ^{1}H NMR (300 MHz, $CDCl_3$) δ 7.35 (s, 2H), 7.28–7.14 (m, 2H), 6.94 (tt, J = 2.3, 8.5 Hz, 1H), 6.27 (br t, J = 5.5 Hz, 1H), 4.04 (t, J = 6.5 Hz, 2H), 3.63 (dt≈q, J = 6.7 Hz, 2H), 2.84 (t, J = 7.0 Hz, 2H), 2.55 (t, J = 7.4 Hz, 2H), 2.27 (s, 6H), 2.10–1.98 (m, 2H); ^{13}C NMR (75 MHz, $CDCl_3$) δ 165.4 (t, $^4J_{C,F}$ = 2.9 Hz), 163.1 (dd, $^3J_{C,F}$ = 12.1 Hz, $^1J_{C,F}$ = 251.0 Hz), 152.3, 137.9 (t, $^3J_{C,F}$ = 8.3 Hz), 137.3, 133.0, 118.6, 110.3 (m_c), 107.1 (t, $^2J_{C,F}$ = 25.3 Hz), 72.2, 56.5, 45.6, 41.3, 34.5, 28.4; HRMS (TOF-ESI+): calcd for $C_{20}H_{23}Br_2F_2N_2O_2$ $[M+H]^+$: 519.0094, found: 519.0090.

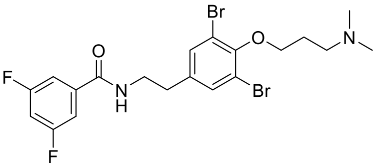

***N*-[3,5-Dibromo-4-[3-(dimethylamino)propoxy]phenethyl]-2-fluorobenzamide (19)**

A white solid (83.7 mg, 62% yield). Mp. 70-72 °C R_f 0.58 (DCM/MeOH, 4:1). ^{1}H NMR (300 MHz, $CDCl_3$) δ 8.08 (ddd, J = 1.8, 7.8, 7.9 Hz, 1H), 7.46 (m, 1H), 7.38 (s, 2H), 7.26 (ddd, J = 1.1, 7.8, 7.6 Hz, 1H), 7.10 (ddd, J = 1.1, 8.3, 12.2 Hz, 1H), 6.83–6.69 (m, 1H), 4.05 (t, J = 6.5 Hz, 2H), 3.72–3.64 (m, 2H), 2.85 (t, J = 7.1 Hz, 2H), 2.55 (t, J = 7.4 Hz, 2H), 2.28 (s, 6H), 2.10–1.99 (m, 2H); ^{13}C NMR (75 MHz, $CDCl_3$) δ 163.5 (d, $J_{C,F}$ = 3.3 Hz), 160.8 (d, $J_{C,F}$ = 247.5Hz), 152.2, 137.5, 133.5 (d, $J_{C,F}$ = 9.2 Hz), 133.0, 132.1 (d, $J_{C,F}$ = 2.2 Hz), 125.0 (d, $J_{C,F}$ = 3.4 Hz), 121.0 (d, $J_{C,F}$ = 11.6 Hz), 118.5, 116.2 (d, $J_{C,F}$ = 24.9 Hz), 72.1, 56.5, 45.6, 41.2, 34.6, 28.4; HRMS (TOF-ESI+): calcd for $C_{20}H_{24}Br_2FN_2O_2$ $[M+H]^+$: 501.0189, found: 501.0193.

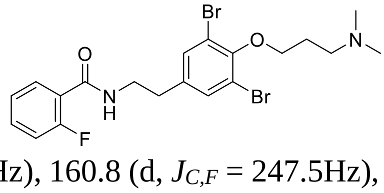

N-[3,5-Dibromo-4-[3-(dimethylamino)propoxy]phenethyl]-3,5-bis(trifluoromethyl)benzamide (20)

A light yellow solid (56.0 mg, 30% yield). Mp. 96.5-98 °C R_f 0.49 (*n*-hexane/acetone, 3:2 + 3% Et_3N). ^1H NMR (300 MHz, $CDCl_3$) δ 8.16 (s, 2H), 8.01 (s, 1H), 7.38 (s, 2H), 6.36 (t, J = 5.6 Hz, 1H), 4.04 (t, J = 6.4 Hz, 2H), 3.69 (q, J = 6.9 Hz, 2H), 2.88 (t, J = 7.0 Hz, 2H), 2.55 (t, J = 7.4 Hz, 2H), 2.28 (s, 6H), 2.12–1.96 (m, 2H); ^{13}C NMR (75 MHz, $CDCl_3$) δ 164.9, 152.4, 137.1., 136.6, 133.0, 132.5 (q, $^2J_{C,F}$ = 34.0 Hz), 127.4 (q, $^3J_{C,F}$ =3.3 Hz), 125.3 (q, $^3J_{C,F}$ =3.6 Hz), 123.0 (q, $^1J_{C,F}$ =273.1 Hz), 118.7, 72.20, 56.5, 45.7, 41.5, 34.4, 29.9, 28.5; HRMS (TOF-ESI+): calcd for $C_{22}H_{23}Br_2F_6N_2O_2$ [M+H]$^+$: 619.0030, found: 6190.0032.

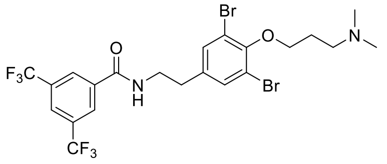

3-[2,6-Dibromo-4-[2-(3-bromo-4-methoxybenzamido)ethyl]phenoxy]-N,N-dimethylpropan-1-aminium chloride (25)

Synthesis of starting material 3-bromo-4-methoxybenzoic acid was done by bromination of 4-methoxybenzoic acid. Yellow oil (104 mg, 67%). ^1H NMR (300 MHz, $CDCl_3$) δ 7.91 (d, J = 2.2 Hz, 1H), 7.67 (dd, J = 8.6, 2.2 Hz, 1H), 7.37 (s, 2H), 6.91 (d, J = 8.6 Hz, 1H), 6.07 (s, 1H), 4.06 (t, J = 6.5 Hz, 2H), 3.94 (s, 3H), 3.64 (q, J = 7.0 Hz, 2H), 2.84 (t, J = 7.0 Hz, 2H), 2.57 (t, J = 7.4 Hz, 2H), 2.29 (s, 6H), 2.14–1.98 (m, 2H).

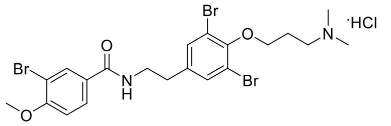

The hydrochloride salt was made by dissolving the free base to 1,4-dioxane (10 mL) and adding a 4 M solution of HCl in 1,4-dioxane (0.5 mL) to this solution. The precipitated hydrochloride salt was filtered and washed with Et_2O. A white solid (44.8 mg, 24.7%). ^1H NMR (300 MHz, CD_3OD) δ 7.97 (t, J = 2.2 Hz, 1H), 7.77 (dd, J = 2.2, 8.6 Hz, 1H), 7.51 (s, 2H), 7.09 (d, J = 8.7 Hz, 1H), 4.12 (t, J = 5.6 Hz, 2H), 3.94 (s, 3H), 3.58–3.47 (m, 4H), 2.97 (s, 6H), 2.86 (t, J = 7.1 Hz, 2H), 2.34–2.23 (m, 2H); ^{13}C NMR (75 MHz, CD_3OD) δ 168.5, 160.0, 152.2, 140.6, 134.5, 133.4, 129.3, 129.0, 118.7, 112.7, 112.4, 71.2, 57.2, 57.0, 43.7, 42.0, 35.2, 26.4; HRMS (TOF-TOF-ESI++): calcd for $C_{21}H_{26}N_2O_3Br_3$ [M+H]$^+$: 590.9494, found: 590.9496.

3,5-Dibromo-N-[3,5-dibromo-4-[3-(dimethylamino)propoxy]phenethyl]-4-methoxybenzamide (26)

Synthesis of starting material 3,5-dibromo-4-methoxybenzoic acid **48** is described on page 14. Recrystallized from *n*-hexane/MeOH. A white solid (54 mg, 31%). Mp. 83-85 °C. ^1H NMR (300 MHz, $CDCl_3$) δ 7.86 (s, 2H), 7.36 (s, 2H), 6.11 (s, 1H), 4.06 (t, J = 6.4 Hz, 2H), 3.92 (s, 3H), 3.63 (q, J = 6.7 Hz, 2H), 2.84 (t, J = 7.0 Hz, 2H), 2.58 (t, J = 7.4 Hz, 2H), 2.30 (s, 6H), 2.06 (m, 2H); ^{13}C NMR (75 MHz, $CDCl_3$) δ 164.7, 157.1, 152.3, 137.4, 133.1, 132.8, 131.6, 118.7, 118.65, 72.1, 60.9, 56.5, 45.6, 41.3, 34.5, 28.3; HRMS (TOF-ESI+): calcd for $C_{21}H_{25}N_2O_3Br_4$ [M+H]$^+$: 668.8599, found: 668.8609.

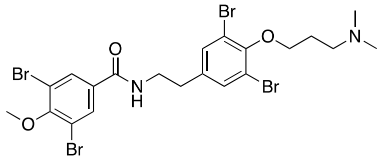

N-[3,5-Dibromo-4-[3-(dimethylamino)propoxy]phenethyl]-4-methoxybenzamide (21)

A white solid (0.100 g, 74% yield). Mp. 106-109 °C. R_f 0.25 (n-hexane/acetone, 3:2 + 3% Et_3N). ^1H NMR (300 MHz, $CDCl_3$) δ 7.71–7.65 (m, 2H), 7.37 (s, 2H), 6.94–6.88 (m, 2H), 6.15 (br t, J = 5.8 Hz, 1H), 4.05 (t, J = 6.3 Hz, 2H), 3.84 (s, 3H), 3.63 (q, J = 6.7 Hz, 2H), 2.84 (t, J = 7.0 Hz, 2H), 2.73 (t, J = 7.5 Hz, 2H), 2.40 (s, 6H), 2.18–2.07 (m, 2H); ^{13}C NMR (75 MHz, $CDCl_3$) δ 167.3, 162.4, 151.9, 138.9, 133.1, 128.8, 126.8, 118.4, 114.0, 71.6, 56.4, 55.6, 45.2, 41.0, 34.8, 27.8.

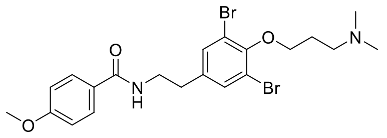

N-[3,5-Dibromo-4-[3-(dimethylamino)propoxy]phenethyl]-5-methylisoxazole-3-carboxamide (22)

A white solid, recrystallized from n-hexane (16.8 mg, 13%). ^1H NMR (300 MHz, $CDCl_3$) δ 7.36 (s, 2H), 6.86 (t, J = 5.4 Hz, 1H), 6.43 (q, J = 0.8 Hz, 1H), 4.04 (t, J = 6.5 Hz, 2H), 3.68–3.52 (m, 2H), 2.82 (t, J = 7.3 Hz, 2H), 2.57–2.50 (m, 2H), 2.47 (d, J = 0.9 Hz, 3H), 2.27 (s, 6H), 2.09–1.97 (m, 2H). ^{13}C NMR (75 MHz, $CDCl_3$) δ 171.4, 159.4, 158.7, 152.2, 137.1, 133.0, 118.6, 101.5, 72.1, 56.5, 45.7, 40.5, 34.6, 28.5, 12.5; HRMS (TOF-ESI+): calcd for $C_{18}H_{24}Br_2N_3O_3$ $[M+H]^+$: 488.0184, found: 488.0184.

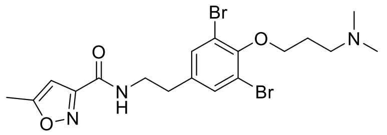

Method B

A 5-mL Biotage tube was charged with corresponding carboxylic acid (2 equiv), EDC•HCl (1.5 equiv) and HOBt (1.5 equiv). The amine (0.10 g, 0.26 mmol) was then added in DCM (1 mL), followed by DIPEA (1.5 equiv) and DCM (3 mL). The resulting mixture was irradiated in MW at 60 °C for 2 h. The reaction mixture was diluted with DCM (10 mL) and washed with a saturated solution of $NaHCO_3$ in H_2O (2 × 10 mL). The aqueous layer was back-extracted in DCM (15 mL), the combined organic layers were dried over anhydrous Na_2SO_4 and concentrated under reduced pressure. The crude product was purified by flash chromatography by using n-hexane (1% TEA)/ EtOAc, gradient 30→80%.

3-Chloro-N-[3,5-dibromo-4-[3-(dimethylamino)propoxy]phenethyl]-4-methoxybenzamide (5)

A white solid (64 mg, 42%). Mp. 79-81 °C, ^1H NMR (300 MHz, $CDCl_3$) δ 7.74 (s, 1H), 7.63-7.60 (m, 1H), 7.33 (s, 2H), 6.94 (d, J = 8.7 Hz, 1H), 6.08 (br s, 1H), 4.04 (t, J = 6.3 Hz, 2H), 3.95 (s, 3H), 3.63 (q, J = 6.3 Hz, 2H), 2.83 (t, J = 6.9 Hz, 2H), 2.54 (t, J = 7.2 Hz, 2H), 2.27 (s, 6H), 2.09-1.99 (m, 2H); ^{13}C NMR (75 MHz, $CDCl_3$) δ 166.1, 157.6, 152.1, 137.6, 133.0, 129.1, 127.5, 127.0, 122.8, 118.5, 111.6, 72.1, 56.5, 45.6, 41.1, 34.6, 28.4; HRMS (TOF-ESI+): calcd for $C_{21}H_{26}N_2O_3ClBr_2$ $[M+H]^+$, 546.9999; found, 546.9999.

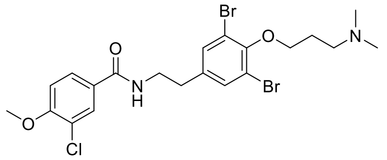

3,5-Dichloro-*N*-[3,5-dibromo-4-[3-(dimethylamino)propoxy]phenethyl]-4-methoxybenzamide (23)

A white solid (85 mg, 59%). Mp. 91-95 °C. ^{1}H NMR (300 MHz, $CDCl_3$) δ 7.65 (s, 2H), 7.34 (s, 2H), 6.11 (br s, 1H), 4.04 (t, J = 6.6 Hz, 2H), 3.93 (s, 3H), 3.62 (q, J = 6.9 Hz, 2H), 2.83 (t, J = 6.9 Hz, 2H), 2.55 (t, J = 7.2 Hz, 2H), 2.28 (s, 6H), 2.09-1.99 (m, 2H); ^{13}C NMR (75 MHz, $CDCl_3$) δ 164.8, 154.9, 152.0, 137.1, 132.8, 131.4, 129.8, 127.6, 118.4, 71.9, 60.8, 56.3, 45.5, 41.1, 34.3, 28.2; HRMS (TOF-ESI+): calcd for $C_{21}H_{25}N_2O_3Cl_2Br_2$ [M+H]$^+$, 580.9609; found, 580.9609.

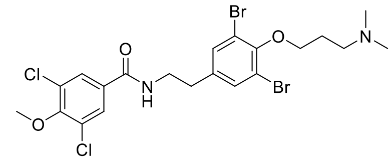

3,5-Dichloro-*N*-[3,5-dibromo-4-[3-(dimethylamino)propoxy]phenethyl]-4-ethoxybenzamide (24)

Synthesis of starting material 2,6-dichloro-4-ethoxybenzoic acid (**51**) is described on page 15. A white solid (130 mg, 82%). Mp. 73-75 °C. ^{1}H NMR (300 MHz, $CDCl_3$) δ 7.64 (s, 2H), 7.36 (s, 2H), 6.11 (br s, 1H), 4.13 (q, J = 6.9 Hz, 2H), 4.04 (t, J = 6.9 Hz, 2H), 3.63 (q, J = 4.5 Hz, 2H), 2.54 (t, J = 7.2 Hz, 2H), 2.27 (s, 6H), 2.09–1.99 (m, 2H), 1.46 (t, J = 7.2 Hz, 2H); ^{13}C NMR (75 MHz, $CDCl_3$) δ 165.05, 154.4, 152.2, 137.3, 133.0, 131.3, 130.1, 127.7, 118.6, 72.1, 70.1, 56.5, 45.7, 41.2, 34.5, 28.4, 156.5; HRMS (TOF-ESI+): calcd for $C_{22}H_{27}N_2O_3Cl_2Br_2$ [M+H]$^+$, 594.9765; found, 594.9764.

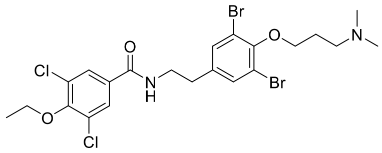

***N*-[3,5-Dibromo-4-[3-(dimethylamino)propoxy]phenethyl]-3-fluoro-4-methoxybenzamide (27)**

A white solid (0.080 g, 58%). Mp. 83-89 °C. ^{1}H NMR (300 MHz, $CDCl_3$) δ 7.47 (t, J = 12.9 Hz, 2H), 7.36 (s, 2H), 6.96 (t, J = 9 Hz, 1H), 6.10 (br s, 1H), 4.04 (t, J = 5.7 Hz, 2H), 3.92 (s, 3H), 3.66–3.58 (m, 2H), 2.83 (t, J = 6.9 Hz, 2H), 2.55 (t, J = 7.2 Hz, 2H), 2.28 (s, 6H), 2.09–2.02 (m, 2H); ^{13}C NMR (75 MHz, $CDCl_3$) δ 166.2, 153.7, 152.1, 150.4, 137.6, 133.0, 127.1, 123.4, 118.5, 115.3, 115.0, 112.8, 72.0, 56.5, 56.4, 45.6, 41.1, 34.6, 28.3; HRMS (TOF-ESI+): calcd for $C_{21}H_{26}N_2O_3FBr_2$ [M+H]$^+$, 531.0294; found, 531.0294.

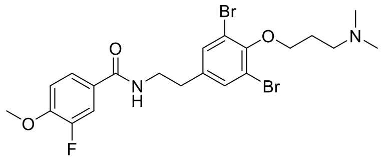

***N*-[3,5-Dibromo-4-[3-(dimethylamino)propoxy]phenethyl]-3,5-difluoro-4-methoxybenzamide (28)**

A white solid (102 mg, 72%). Mp. 93-96 °C. ^{1}H NMR (300 MHz, $CDCl_3$) δ 7.32 (s, 2H), 7.23 (s, 2H), 6.06 (br s, 1H), 4.04–3.99 (m, 4H), 3.59 (q, J = 6.6 Hz, 2H), 2.80 (t, J = 6.9 Hz, 2H), 2.53 (t, J = 7.2 Hz, 2H), 2.25 (s, 6H), 2.06–1.97 (m, 2H); ^{13}C NMR (75 MHz, $CDCl_3$) δ 165.1, 156.8, 156.7, 153.5, 153.4, 152.1, 137.4, 133.0, 128.6, 118.5, 111.5, 111.4, 111.3, 111.2, 72.0, 61.8, 56.4, 45.5, 41.2, 34.5, 28.3; HRMS (TOF-ESI+): calcd for $C_{21}H_{25}N_2O_3F_2Br_2$ [M+H]$^+$, 549.0200; found, 549.0200.

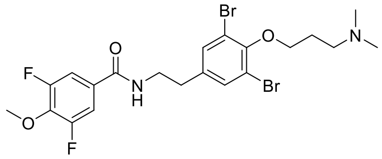

***N*-[3,5-Dibromo-4-[3-(dimethylamino)propoxy]phenethyl]-3-iodo-4-methoxybenzamide (29)**

A white solid (0.062 g, 37%). ^{1}H NMR(300 MHz, $CDCl_3$) δ 8.11 (s, 1H), 7.71 (d, J = 8.7 Hz, 1H), 7.36 (s, 2H), 6.82 (d, J = 8.7 Hz, 1H), 6.08 (br s, 1H), 4.04 (t, J = 6.6 Hz, 2H), 3.92 (s, 3H), 3.62 (q, J = 6.6 Hz, 2H), 2.83 (t, J = 6.9 Hz, 2H), 2.54 (t, J = 7.5 Hz, 2H), 2.27 (s, 6H), 2.08–1.99 (m, 2H); ^{13}C NMR (75 MHz, $CDCl_3$) δ 165.8, 160.7, 152.1, 138.2, 137.6, 133.0,

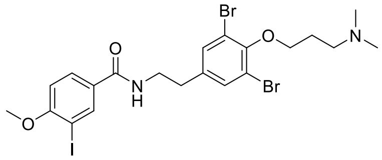

128.9, 128.6, 118.5, 110.3, 85.9, 72.1, 56.7, 56.5, 45.6, 41.1, 34.6, 28.4; HRMS (TOF-ESI+): calcd for $C_{21}H_{26}N_2O_3IBr_2$ $[M+H]^+$, 638.9355; found, 638.9355.

N-[3,5-Dibromo-4-[3-(dimethylamino)propoxy]phenethyl]-4-methoxy-3-methylbenzamide (30)

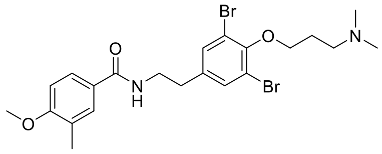

A light yellow oil (71 mg, 51%). ^{1}H NMR (300 MHz, $CDCl_3$) δ 7.56 (d, J = 7.5 Hz, 1H), 7.50 (s, 1H), 7.73 (s, 2H), 6.82 (d, J = 8.4 Hz, 1H), 6.06 (br s, 1H), 4.04 (t, J = 6.3 Hz, 2H), 3.86 (s, 3H), 3.63 (q, J = 6.3 Hz, 2H), 2.83 (t, J = 6.9 Hz, 2H), 2.56 (t, J = 7.5 Hz, 2H), 2.28 (s, 6H), 2.23 (s, 3H), 2.09–2.00 (m, 2H); ^{13}C NMR (75 MHz, $CDCl_3$) δ 167.5, 160.5, 152.0, 137.8, 133.0, 129.3, 126.9, 126.3, 118.5, 110.1, 109.5, 72.0, 56.5, 55.6, 45.6, 40.9, 34.8, 28.4, 16.4; HRMS (TOF-ESI+): calcd for $C_{22}H_{29}N_2O_3Br_2$ $[M+H]^+$, 527.0545; found, 527.0545.

N-[3,5-Dibromo-4-[3-(dimethylamino)propoxy]phenethyl]-3,4-dimethoxybenzamide (31)

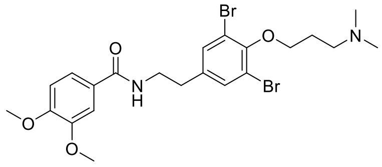

A pale yellow oil (61 mg, 42%). ^{1}H NMR (300 MHz, $CDCl_3$) δ 7.38 (s, 3H), 7.18 (d, J = 8.4 Hz, 1H), 6.85 (d, J = 8.1 Hz, 1H), 6.06 (br s, 1H), 4.05 (t, J = 6.3 Hz, 2H), 3.93 (s, 3H), 3.91 (s, 3H), 3.62 (q, J = 7.2 Hz, 2H), 2.85 (t, J = 6.9 Hz, 2H), 2.57 (t, J = 6.6 Hz, 2H), 2.29 (s, 6H), 2.10–2.01 (m, 2H); ^{13}C NMR (75 MHz, $CDCl_3$) δ 167.3, 152.0, 151.9, 149.1, 137.8, 133.0, 127.1, 119.3, 118.5, 110.5, 110.4, 110.1, 72.0, 56.4, 56.1, 45.6, 41.1, 34.7, 28.3; HRMS (TOF-ESI+): calcd for $C_{22}H_{29}N_2O_4Br_2$ $[M+H]^+$, 543.0494; found, 543.0494.

N-(3-Chloropropyl)-2,2,2-trifluoro-*N*-methylacetamide (36)

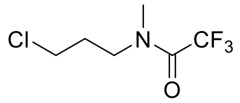

3-Chloro-*N*-methylpropan-1-amine hydrochloride (1.00 g, 6.94 mmol) was dissolved to dry DCM (10 mL). The reaction mixture was cooled and trifluoroacetic anhydride (1.16 mL, 8.33 mmol, 1.2 equiv) and triethylamine (1.95 mL, 13.9 mmol, 2.0 equiv) were added. The resulting mixture was stirred for 1 h, let to warm to rt, stirred further for 20 h and monitored by TLC (*n*-hexane: EtOAc 4:1). The reaction mixture was concentrated *in vacuo* to give crude **36**, which was purified by flash chromatography (eluent: cyclohexane-EtOAc; gradient 0→30%). Compound **36** was obtained as a colorless liquid (1.16 g, 82%). ^{1}H NMR (300 MHz, $CDCl_3$) δ 3.66-3.55 (m, 4H), 3.17 (s, 2H), 3.05 (s, 1H), 2.15-2.05 (m, 2H); ^{13}C NMR(75 MHz, $CDCl_3$) δ 157.8, 157.4, 157.3, 157.1, 156.9, 156.7, 120.9, 120.8, 118.0, 117.9, 115.2, 115.1, 112.3, 112.2, 47.6, 47.4, 42.1, 41.6, 35.7, 35.6, 34.9, 31.2, 29.6.

tert-Butyl [3,5-dibromo-4-[3-(2,2,2-trifluoro-*N*-ethylacetamido)propoxy]phenethyl]carbamate (37)

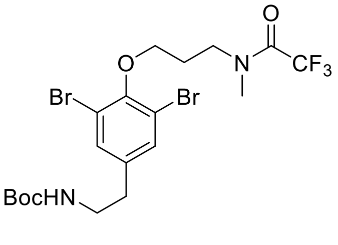

Compound **8** (1.20 g, 3.04 mmol), Cs_2CO_3 (1.19 g, 3.65 mmol, 1.2 equiv) and **36** (0.74 g, 3.65 mmol, 1.2 equiv) were dissolved in DMF (10 mL) under argon atmosphere. The reaction mixture was heated at 80 °C for 14 h. The reaction mixture was treated with water (20 mL) and extracted with Et_2O (2 × 30 mL). The ether layer was washed with water (20 mL), the combined organic layers ware dried over anhydrous Na_2SO_4 and concentrated. The purification was performed by flash chromatography (eluent: EtOAc:*n*-heptane, gradient, 10→50%). Compound **37** was obtained as a white solid (1.56 g, 92%). Mp. 81-83 °C. ^{1}H NMR (300 MHz, $CDCl_3$) δ 7.33 (s, 2H), 4.53 (br s, 1H), 4.04 (t, J = 6 Hz, 2H), 7.75-3.72 (m, 2H), 3.32 (q, J = 6.6 Hz, 2H), 3.22 (s, 2H), 3.11 (s, 1H), 2.72 (t, J = 6.9 Hz, 2H), 2.21-2.12 (m, 2H); 1.44 (s, 9H); ^{13}C NMR (75 MHz, $CDCl_3$) δ 157.3, 156.9, 155.8, 151.6, 151.4,

138.1, 133.1, 118.1, 79.7, 70.6, 70.1, 47.4, 47.3, 41.6, 35.5, 35.1, 34.8, 29.2, 28.5, 27.3; HRMS (TOF-ESI+): calcd for $C_{19}H_{25}N_2O_4F_3Br_2Na$ [M+Na]$^+$, 583.0031; found, 583.0034.

N-[3-(4-(2-aminoethyl)-2,6-dibromophenoxy]propyl]-2,2,2-trifluoro-*N*-methylacetamide (38)

A solution of **37** (1.20 g, 2.13 mmol) in DCM (10 mL) was stirred with TFA (0.82 mL, 14.3 mmol, 6.0 equiv) for 14 h. The reaction mixture was then quenched with a saturated solution of $NaHCO_3$ in H_2O and extracted with DCM (3 × 10 mL). The organic layer was dried over anhydrous Na_2SO_4 and concentrated *in vacuo* to give **38** as a white solid (1.20 g, quant.). The product was used in the next step without further purification. ^{1}H NMR (300 MHz, CD_3OD) δ 7.54 (s, 2H), 4.08-4.04 (m, 2H), 3.84-3.73 (m, 2H), 3.23 (s, 2H), 3.20-3.14 (m, 2H), 3.10 (s, 1H), 2.90 (t, J = 7.8 Hz, 2H), 2.24-2.13 (m, 2H); ^{13}C NMR (75 MHz, MeOH-d_4) δ 162.8, 158.3, 157.9, 153.4, 153.2, 138.2, 138.1, 134.3, 122.2, 119.2, 116.5, 71.9, 71.6, 71.4, 42.0, 35.6, 34.9, 34.5, 34.2, 29.9, 28.2; HRMS (TOF-ESI+): calcd for $C_{14}H_{18}N_2O_2F_3Br_2$ [M+H]$^+$, 460.9687; found, 460.9687.

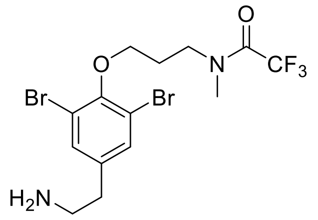

General method for an amide coupling

A 5-mL MW tube was charged with amine (0.1-0.2 g), the corresponding carboxylic acid (1.5 equiv), EDC·HCl (1.5 equiv), HOBt (1.5 equiv) and DIPEA (1.5 equiv) in dry DCM (4 mL). The resulting mixture was MW irradiated in a sealed tube for 1.5 h at 60 °C (p = 5 bar). The reaction mixture was diluted with DCM (10 mL) and washed with a saturated solution of $NaHCO_3$ in H_2O (3 × 10 mL). The aqueous layer was back extracted with DCM (10 mL) and the combined organic layers were then washed with a 2 M solution of HCl in H_2O (20 mL). The DCM layer was then dried over anhydrous Na_2SO_4, filtered and evaporated. The purification was performed by using flash chromatography with an EtOAc:*n*-heptane gradient.

N-[3,5-Dibromo-4-[3-(2,2,2-trifluoro-*N*-methylacetamido)propoxy]phenethyl]-4-methoxybenzamide (39)

A white solid (91 mg, 71%). Mp. 119-122 °C. ^{1}H NMR (300 MHz, $CDCl_3$) δ 7.67 (d, J = 9 Hz, 2H), 6.92 (d, J = 8.7 Hz, 2H), 6.05 (br s, 1H), 4.07-4.03 (m, 2H), 3.85 (s, 3H), 3.77-3.72 (m, 2H), 3.68-3.61 (m, 2H), 3.23 (s, 2H), 3.11 (s, 1H), 2.85 (t, J = 7.5 Hz, 2H), 2.22-2.13 (m, 2H); ^{13}C NMR (75 MHz, $CDCl_3$) δ 167.2, 162.4, 138.3, 138.2, 133.1, 128.7, 118.3, 114.0, 70.7, 70.2, 55.6, 47.4, 41.0, 34.8, 29.3, 27.4; HRMS (TOF-ESI+): calcd for $C_{22}H_{24}N_2O_4F_3Br_2$ [M+H]$^+$, 595.0055; found, 595.0055.

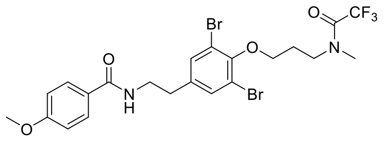

3-Chloro-*N*-[3,5-dibromo-4-[3-(2,2,2-trifluoro-*N*-methylacetamido)propoxy]phenethyl]-4-methoxybenzamide (40)

A white solid (0.18 g, 68%). Mp. 87 °C. ^{1}H NMR (300 MHz, $CDCl_3$) δ 7.74 (s, 1H), 7.62 (d, J = 9.9 Hz, 1H), 7.38 (s, 2H), 6.94 (d, J = 8.4 Hz, 1H), 6.03 (br s, 1H), 4.05 (t, J = 6 Hz, 2H), 3.95 (s, 3H), 3.81-3.72 (m, 2H), 3.65-3.63 (m, 2H), 3.22 (s, 2H), 3.11 (s, 1H), 2.85 (t, J = 6.6 Hz, 2H), 2.19-2.14 (m, 2H); ^{13}C NMR (75 MHz, $CDCl_3$) δ 166.1, 157.8, 151.9, 138.1, 138.0, 133.2, 129.2, 129.2, 127.7, 127.1, 123.0, 118.4, 118.4, 111.7, 77.2,

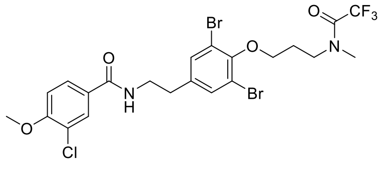

70.7, 70.2, 56.5, 47.4, 41.1, 35.5, 34.7, 29.3, 27.4; HRMS (TOF-ESI+): calcd for $C_{22}H_{23}Br_2ClF_3N_2O_4$ $[M+H]^+$, 628.9665; found, 628.9665.

3,5-Dichloro-*N*-[3,5-dibromo-4-[3-(2,2,2-trifluoro-*N*-methylacetamido)propoxy]phenethyl]-4-methoxybenzamide (41)

A white solid (0.16 g, 56%). Mp. 94-99 °C. ^{1}H NMR (300 MHz, $CDCl_3$) δ 7.65 (s, 2H), 7.37 (s, 2H), 6.09 (br s, 1H), 4.08-3.94 (m, 2H), 3.79 (s, 3H), 3.79-3.75 (m, 2H), 3.72-3.60 (m, 2H), 3.22 (s, 2H), 3.11 (s, 1H), 2.85 (t, J = 8.4 Hz, 2H), 2.12-2.15 (m, 2H); ^{13}C NMR (75 MHz, $CDCl_3$) δ 165.0, 155.2, 151.9, 151.7, 137.8, 137.7, 133.1, 131.6, 130.0, 127.8, 118.4, 70.7, 70.2, 61.0, 47.4, 41.3, 35.4, 34.8, 34.6, 29.3, 27.4; HRMS (TOF-ESI+): calcd for $C_{22}H_{22}N_2O_4Cl_2F_3Br_2$ $[M+H]^+$, 662.9275; found, 662.9276.

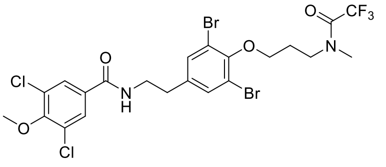

N-[3,5-Dibromo-4-[3-(2,2,2-trifluoro-*N*-methylacetamido)propoxy]phenethyl]-3,5-difluoro-4-methoxybenzamide (42)

A white solid (0.18 g, 66%). Mp. 124-127 °C. ^{1}H NMR (d_6-DMSO, 300 MHz) δ 8.56 (br s, 1H), 7.57-7.53 (m, 4H), 3.99 (s, 3H), 3.96-3.92 (m, 2H), 3.73-3.61 (m, 2H), 3.49-3.43 (m 2H), 3.29 (s, 3H), 3.15 (s, 2H), 3.02 (s, 1H), 2.79 (t, J = 7.8 Hz, 2H), 2.11-2.04 (m, 2H); ^{13}C NMR (75 MHz, $CDCl_3$) δ 52.6, 150.6, 139.0, 133.0, 129.0, 117.1, 111.7, 111.4, 70.8, 70.4, 61.7, 46.4, 34.6, 34.1, 33.2, 28.4, 26.6; HRMS (TOF-ESI+): calcd for $C_{22}H_{22}N_2O_4F_5Br_2$ $[M+H]^+$, 630.9866; found, 630.9866.

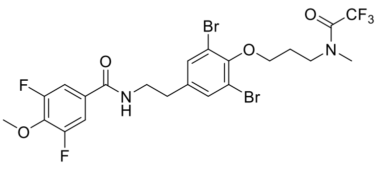

General method for 2,2,2-trifluoro-*N*-methylacetamido cleavage

A solution of trifluoroacetyl-protected compound (0.080 g, 0.13 mmol), K_2CO_3 (3.0 equiv) in MeOH (3 mL) and water (1 mL) was refluxed for 3 h. The reaction was monitored by TLC (*n*-hexane/Et_3N, 1%): acetone 3:2). The reaction mixture was concentrated *in vacuo*. The residue was partitioned between EtOAc (10 mL) and water (5 mL) and the organic layer was separated. The aqueous layer was then extracted again with EtOAc (2 × 5 mL). The combined organic layers were dried over anhydrous Na_2SO_4 and evaporated.

N-[3,5-Dibromo-4-[3-(methylamino)propoxy]phenethyl]-4-methoxybenzamide (43)

A white solid (0.063 g, 94%). Mp. 89-97 °C. ^{1}H NMR (d_6-DMSO, 300 MHz) δ 8.35 (br s, 1H), 7.76 (d, J = 8.4 Hz, 2H), 7.51 (s, 2H), 6.98 (d, J = 8.7 Hz, 2H), 3.96 (t, J = 6.3 Hz, 2H), 3.80 (s, 3H), 3.48-3.42 (m, 2H), 2.81-2.77 (m, 2H), 2.65 (t, J = 6.6 Hz, 2H), 2.28 (s, 3H), 1.93-1.84 (m, 2H); ^{13}C NMR (75 MHz, $CDCl_3$) δ 167.2, 162.4, 152.0, 137.8, 133.1, 128.7, 126.8, 118.5, 114.0, 72.2, 55.6, 49.2, 41.0, 36.7, 34.8, 30.3; HRMS (TOF-ESI+): calcd for $C_{20}H_{25}N_2O_3Br_2$ $[M+H]^+$, 499.0232; found, 499.0232.

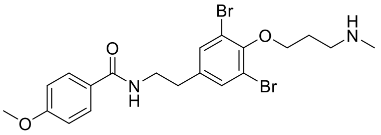

3-Chloro-*N*-[3,5-dibromo-4-[3-(methylamino)propoxy]phenethyl]-4-methoxybenzamide (CB-44)

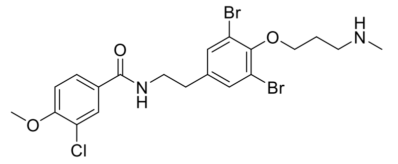

A pale yellow oil (0.083 g, 65%). ^{1}H NMR (300 MHz, $CDCl_3$) δ 7.74 (s, 1H), 7.61 (d, J = 9.9 Hz, 1H), 7.37 (s, 2H), 6.94 (d, J = 8.7 Hz, 1H), 6.04 (s, 1H), 4.08 (t, J = 6 Hz, 2H), 3.94 (s, 3H), 3.64 (q, J = 7.2 Hz, 2H), 2.92 (t, J = 6.3 Hz, 2.84 (s, J = 6.9 Hz, 2H), 2.51 (s, 3H), 2.12-2.00 (m, 3H); ^{13}C NMR (75 MHz, $CDCl_3$) δ 166.3, 157.6, 151.8, 137.7, 132.9, 129.1, 127.6, 127.1, 122.7, 118.3, 111.6, 72.1, 56.4, 49.1, 41.1, 36.5, 34.6, 30.1; HRMS (TOF-ESI+): calcd for $C_{20}H_{24}N_2O_3ClBr_2$ $[M+H]^+$, 532.9842; found, 532.9842.

3,5-Dichloro-*N*-[3,5-dibromo-4-[3-(methylamino)propoxy]phenethyl]-4-methoxybenzamide (45)

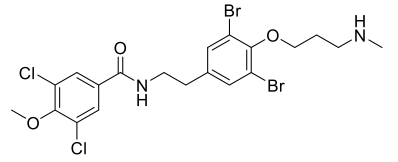

A white solid (0.091 g, 99%). Mp. 86-97 °C. ^{1}H NMR (300 MHz, $CDCl_3$) δ 7.65 (s, 2H), 7.37 (s, 2H), 6.07 (br s, 1H), 4.08 (t, J = 6.3 Hz, 2H), 3.94 (s, 3H), 3.63 (q, J = 7.2 Hz, 2H), 2.94-2.82 (m, 4H), 2.50 (s, 3H), 2.12-2.04 (m, 2H), 1.91 (br s, 1H); ^{13}C NMR (75 MHz, $CDCl_3$) δ 164.9, 155.2, 152.2, 137.4, 133.1, 131.7, 130.0, 127.8, 118.6, 77.6, 72.2, 61.0, 49.1, 41.3, 36.5, 34.5, 30.1; HRMS (TOF-ESI+): calcd for $C_{20}H_{23}N_2O_3Cl_2Br_2$ $[M+H]^+$, 566.9452; found, 566.9453.

***N*-[3,5-Dibromo-4-[3-(methylamino)propoxy]phenethyl]-3,5-difluoro-4-methoxybenzamide (46)**

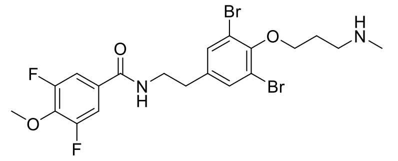

A white solid (0.091 g, 71%). Mp. 87-92 °C. ^{1}H NMR (300 MHz, $CDCl_3$) δ 7.33 (s, 2H), 7.23 (s, 2H), 6.04 (br s, 1H), 4.07-4.03 (m, 5H), 3.64-3.57 (m, 2H), 2.88-2.78 (m, 2H), 2.46 (s, 3H), 2.07-1.99 (m, 2H), 1.71 (br s, 1H); ^{13}C NMR (75 MHz, $CDCl_3$) δ 165.1, 156.9, 153.6, 153.5, 152.2, 137.4, 133.0, 128.7, 118.6, 111.6, 111.4, 111.3, 111.2, 72.2, 61.8, 49.1, 41.2, 36.5, 34.6, 30.2; HRMS (TOF-ESI+): calcd for $C_{20}H_{23}N_2O_3F_2Br_2$ $[M+H]^+$, 535.0043; found, 535.0043.

Synthesis of non-brominated derivative 35

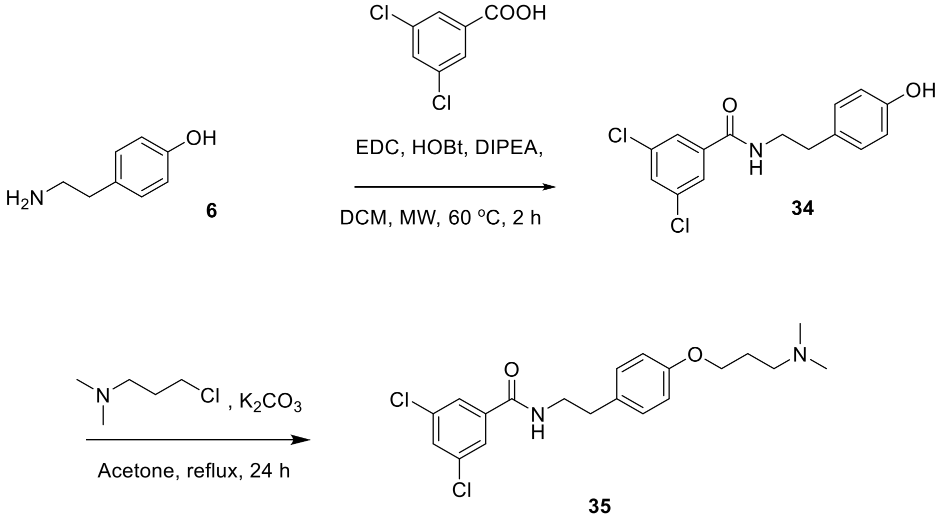

Scheme 1: Synthesis of non-brominated tyramine derivative **35**

3,5-Dichloro-*N*-(4-hydroxyphenethyl)benzamide (34)

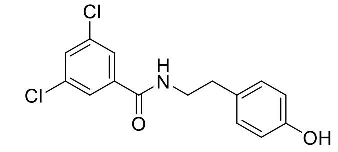

Tyramine **6** (130 mg, 0.95 mmol), 3,5-dichlorobenzoic acid (181 mg, 0.95 mmol) and 2-(1*H*-benzotriazol-1-yl)-1,1,3,3-tetramethyluronium hexafluorophosphate (HBTU, 431 mg, 1.14 mmol, 1.2 equiv) were suspended in dry DCM (10 mL) under argon atmosphere. The resulting mixture was stirred at room temperature for 3 d. The mixture was then diluted with DCM (10 mL) and washed with H_2O (2 × 20 mL) The aqueous phase was re-extracted with DCM (2 × 20 mL). The combined organic phases were dried with anhydrous Na_2SO_4, filtered and the solvent was removed *in vacuo*. The crude product was purified by flash chromatography (eluent: *n*-hexane/EtOAc; gradient 20→40%). The product was recrystallized from DCM/MeOH (40:1) to give a white crystalline solid (87.3 mg, 30%). Mp. 165–169 °C. R_f 0.34 (*n*-hexane/EtOAc, 3:2). ^{1}H NMR (300 MHz, d_6-DMSO) δ 9.16 (s, 1H), 8.73 (t, J = 5.6 Hz, 1H), 7.83 (d, J = 1.9 Hz, 2H), 7.78 (t, J = 1.9 Hz, 1H), 7.11–6.87 (m, 2H), 6.76–6.56 (m, 2H), 3.47–3.35 (m, 2H), 2.72 (t, J = 7.8 Hz, 2H); ^{13}C NMR (75 MHz, d_6-DMSO) δ 163.3, 155.7, 137.8, 134.2, 130.5, 129.5, 129.3, 126.0, 115.1, 41.4, 34.0; HRMS (TOF-ESI+): calcd for $C_{15}H_{14}Cl_2NO_2$ $[M+H]^+$: 310.0402, found: 310.0401.

3,5-Dichloro-*N*-[4-[3-(dimethylamino)propoxy]phenethyl]benzamide (35)

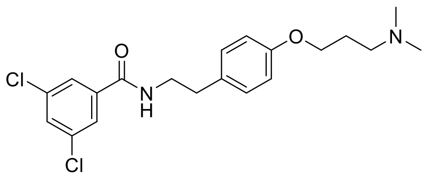

3,5-Dichloro-*N*-(4-hydroxyphenethyl)benzamide **34** (50.0 mg, 0.16 mmol), 3-chloro-*N*,*N*-dimethylpropylamine hydrochloride (30.7 mg, 0.19 mmol, 1.2 equiv) and anhydrous K_2CO_3 (55.7 mg, 0.40 mmol, 2.5 equiv) were suspended in dry acetone (2.5 + 2 mL) under argon atmosphere. The mixture was refluxed for 2 d. The solvent was then removed *in vacuo*. The crude product was extracted to DCM (10 mL) and washed with H_2O (8 mL). The aqueous phase was re-extracted with DCM (2 × 8 mL). The combined organic layers were dried over anhydrous Na_2SO_4, filtered and the solvent was removed *in vacuo* to give the crude product as a white/light yellow solid (73.0 mg). The solid was purified by flash chromatography (eluent: DCM/MeOH; gradient 0→20%) to give a white solid (43.0 mg, 68%). ^{1}H NMR (300 MHz, $CDCl_3$) δ 7.54 (d, J = 1.9 Hz, 2H), 7.47–7.44 (m, 1H), 7.15–7.08 (m, 2H), 6.90–6.83 (m, 2H), 6.14 (br s, 1H), 4.00 (t, J = 6.4 Hz, 2H), 3.70–3.60 (m, 2H), 2.86 (t, J = 6.9 Hz, 2H), 2.52 (t, J = 7.3 Hz, 2H), 2.30 (s, 6H), 2.04–1.92 (m, 2H); ^{13}C NMR (75 MHz, $CDCl_3$) δ 165.0, 158.0, 137.8, 135.6, 131.4, 130.6, 129.8, 125.7, 115.0, 66.3, 56.5, 45.5, 41.7, 34.8, 27.5; HRMS (TOF-ESI+): calcd for $C_{20}H_{25}Cl_2N_2O_2$ $[M+H]^+$: 395.1293, found: 395.1292.

Synthesis of 3,5-dibromo-4-methoxybenzoic acid x

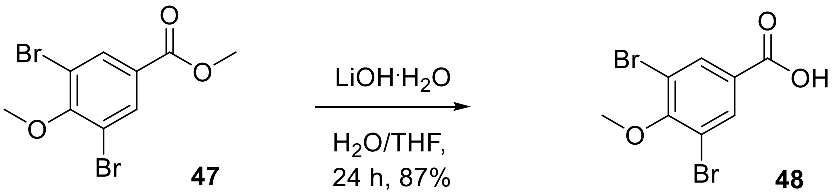

3,5-Dibromo-4-methoxybenzoic acid (48) [4]

A solution of methyl 3,5-dibromo-4-methoxybenzoate (**47**) (0.78 g, 2.41 mmol), synthesized according to Grimster [3], and LiOH·H_2O (0.25 g, 6.01 mmol, 2.5 equiv) in 1:1 H_2O/THF (6 mL) was heated to 60 °C. After 24 h TLC (1:1 *n*-hexane:EtOAc) indicated completion of reaction. A 1 M solution of HCl in H_2O (10 mL) was added and the aqueous phase was extracted with EtOAc (3 × 25

ml). The organic phase was dried over anhydrous Na_2SO_4, filtered and evaporated *in vacuo* to give a white solid (0.64 g, 86%). ^{1}H NMR (300 MHz, d_6-DMSO) δ 13.45 (s, 1H), 8.10 (s, 2H), 3.87 (s, 3H). ^{13}C NMR (75 MHz, d_6-DMSO) δ 164.44, 157.00, 133.54, 129.53, 117.65, 60.58, 39.52.

Synthesis of 2,6-dichloro-4-ethoxybenzoic acid (51)

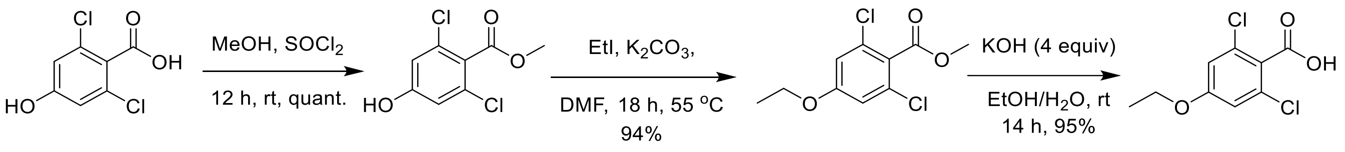

49 **50** **51**

Methyl 2,6-dichloro-4-hydroxybenzoate (49)

To a cooled solution of 3,5-dichloro-4-hydroxybenzoic acid (0.50 g, 2.41 mmol) in MeOH (10 mL), $SOCl_2$ (0.35 mL, 2.0 equiv) was added dropwise. The reaction mixture was stirred under argon atmosphere for 14 h at rt. It was quenched with a saturated solution of $NaHCO_3$ in H_2O (10 mL) and extracted with DCM (25 mL). The organic layer was then washed with a saturated solution of $NaHCO_3$ in H_2O (3 × 10 mL), dried over anhydrous Na_2SO_4 and concentrated to give **49** (0.56 g, quant) as a white amorphous solid. ^{1}H NMR (300 MHz, $CDCl_3$) δ 7.97 (s, 2H), 6.22 (br s, 1H), 3.90 (s, 3H).

Methyl 2,6-dichloro-4-ethoxybenzoate (50)

A mixture of ester **49** (0.50 g, 2.26 mmol), K_2CO_3 (0.92 g, 6.78 mmol, 3.0 equiv) and EtI (0.55 mL, 6.78 mmol, 3.0 equiv) in DMF (10 mL) was flushed with argon and stirred at 55 °C for 18 h. Water (10 mL) was then added to the reaction mixture that was subsequently extracted with Et_2O (3 × 20 mL). The combined organic layers were washed with a saturated solution of $Na_2S_2O_3$ in H_2O (2 × 30 mL), dried over anhydrous Na_2SO_4 and concentrated *in vacuo*. The crude product was purified by flash chromatography (eluent: *n*-hexane-EtOAc; gradient 0→25% to give compound **50** as a white amorphous solid (0.53 g, 94%). ^{1}H NMR (300 MHz, $CDCl_3$) δ 7.97 (s, 2 H), 4.16 (q, J = 7.2 Hz, 2H), 3.91 (s, 3H), 1.48 (t, J = 6.9 Hz, 3H).

2,6-Dichloro-4-ethoxybenzoic acid (51)

A mixture of **50** (0.50 g, 2.01 mmol) and KOH (0.68 g, 12.0 mmol, 6.0 equiv) in MeOH (5 mL) was stirred for 14 h. The reaction mixture was then concentrated *in vacuo* and acidified with a concentrated solution of HCl. The resulting solid was filtered and further dissolved in DCM, dried over anhydrous Na_2SO_4 and concentrated *in vacuo* to give **51** as a white amorphous solid (0.45 g, 95%). Mp. 178-180 °C. ^{1}H NMR (300 MHz, $CDCl_3$) δ 8.04 (s, 2H), 4.18 (q, J = 6.9 Hz, 2H), 1.49 (t, J = 7.2 Hz, 3H). ^{13}C NMR (100 MHz, $CDCl_3$) δ 169.7, 156.5, 131.0, 130.2, 126.0, 70.3, 15.7. (measured using Bruker Avance 400 MHz NMR with smart probe)

2. ^{1}H and ^{13}C NMR spectra of compound 5

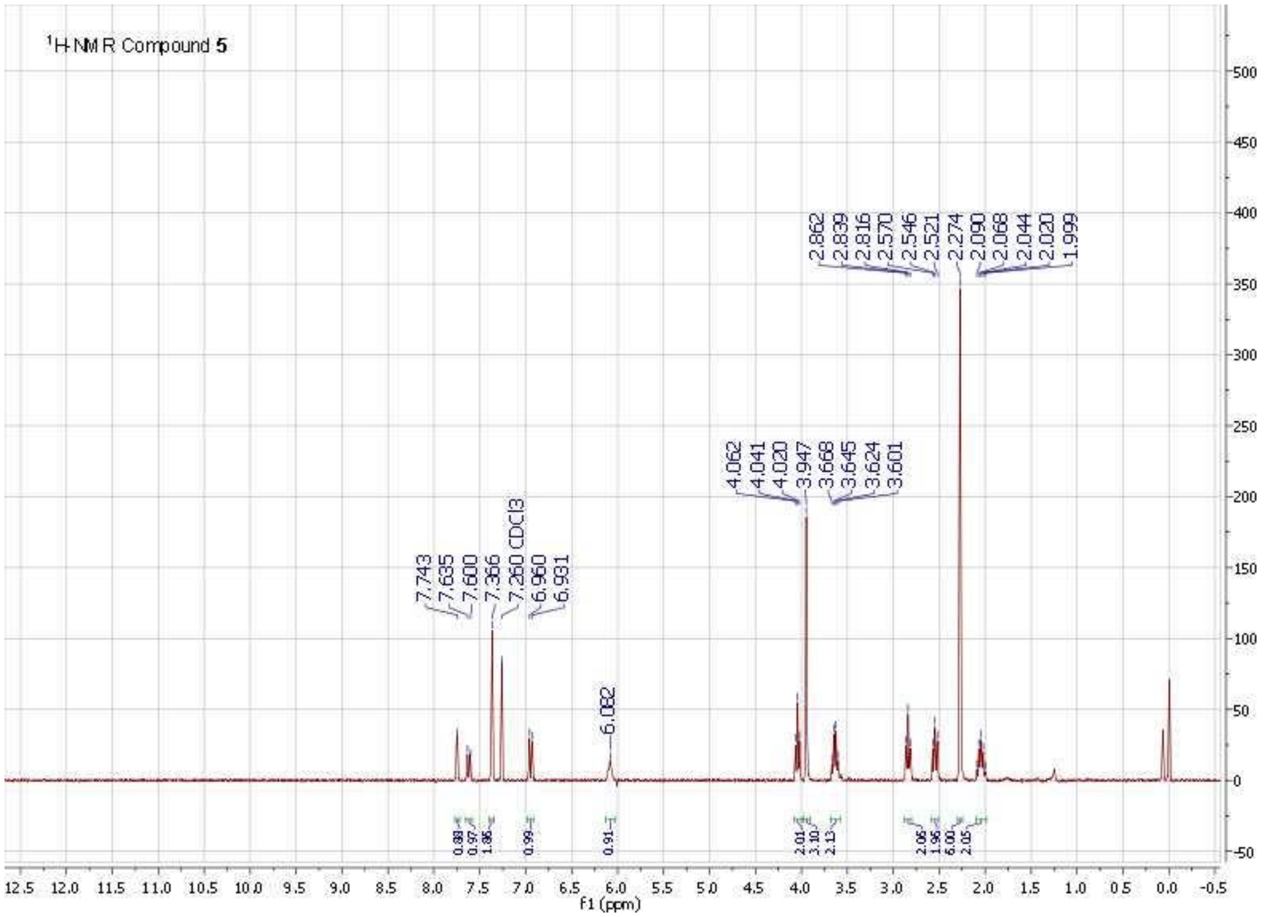

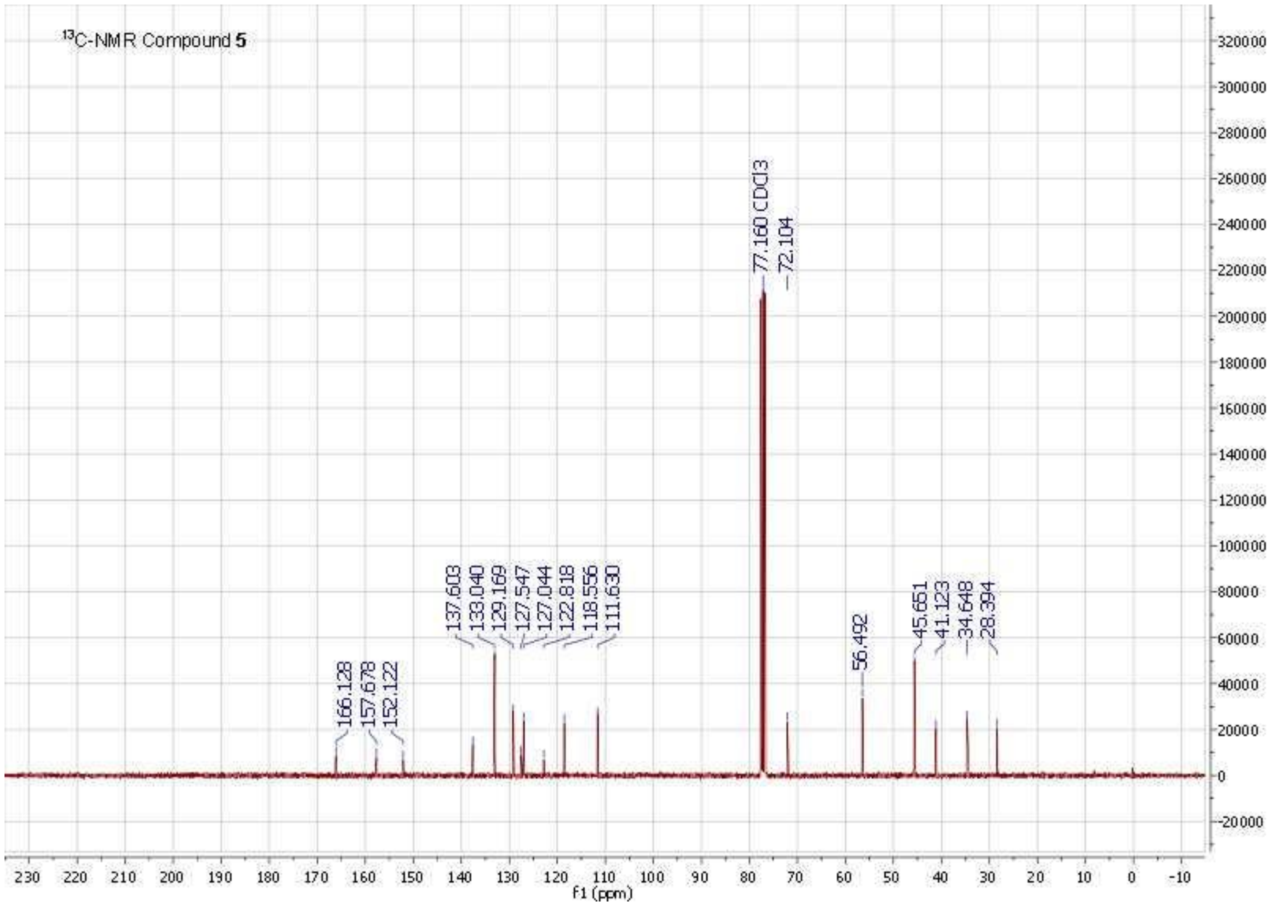

3. References

[1] Yoshida M, Yamaguchi K, Total Synthesis of dispyrin, purpurealidin E, and aplysamine-1. Chem Pharm Bull. 2008;56: 1362-1363.
http://doi.org/10.1248/cpb.56.1362

[2] Kottakota SK, Evangelopoulos D, Alnimr A, Bhakta S, McHugh, TD, Gray M, Groundwater PW, Marrs EL, Perry JD, Spilling CD, Harburn JJ. Synthesis and biological evaluation of purpurealidin E-derived marine sponge metabolites: Aplysamine-2, aplyzanzine A, and suberedamines A and B. J Nat Prod. 2012;75: 1090-1101. DOI: 10.1021/np300102z

[3] Grimster NP, Connelly S, Baranczak A, Dong J, Krasnova LB, Sharpless KB, Powers ET, Wilson IA, Kelly JW. Aromatic sulfonyl fluorides covalently kinetically stabilize transthyretin to prevent amyloidogenesis while affording a fluorescent conjugate. J Am Chem Soc. 2013;135: 5656-5668. DOI: 10.1021/ja311729d

[4] Raju BG, Odowd H, Gao H, Patel DV, Trias J, Inventors; *N*-hydroxyamide derivatives possessing antibacterial activity. WO 2004/007444. 2004 Jan 22
