# Supplementary material for: Synthesis of novel purpurealidin analogs and evaluation of their effect on the cancer-relevant potassium channel KV10.1
Source: PLoS One. 2017 Dec 8;12(12):e0188811. doi: 10.1371/journal.pone.0188811 (PMC5722316; doi:10.1371/journal.pone.0188811)
Supplement: S1 Fig — (PDF) [file pone.0188811.s002.pdf]

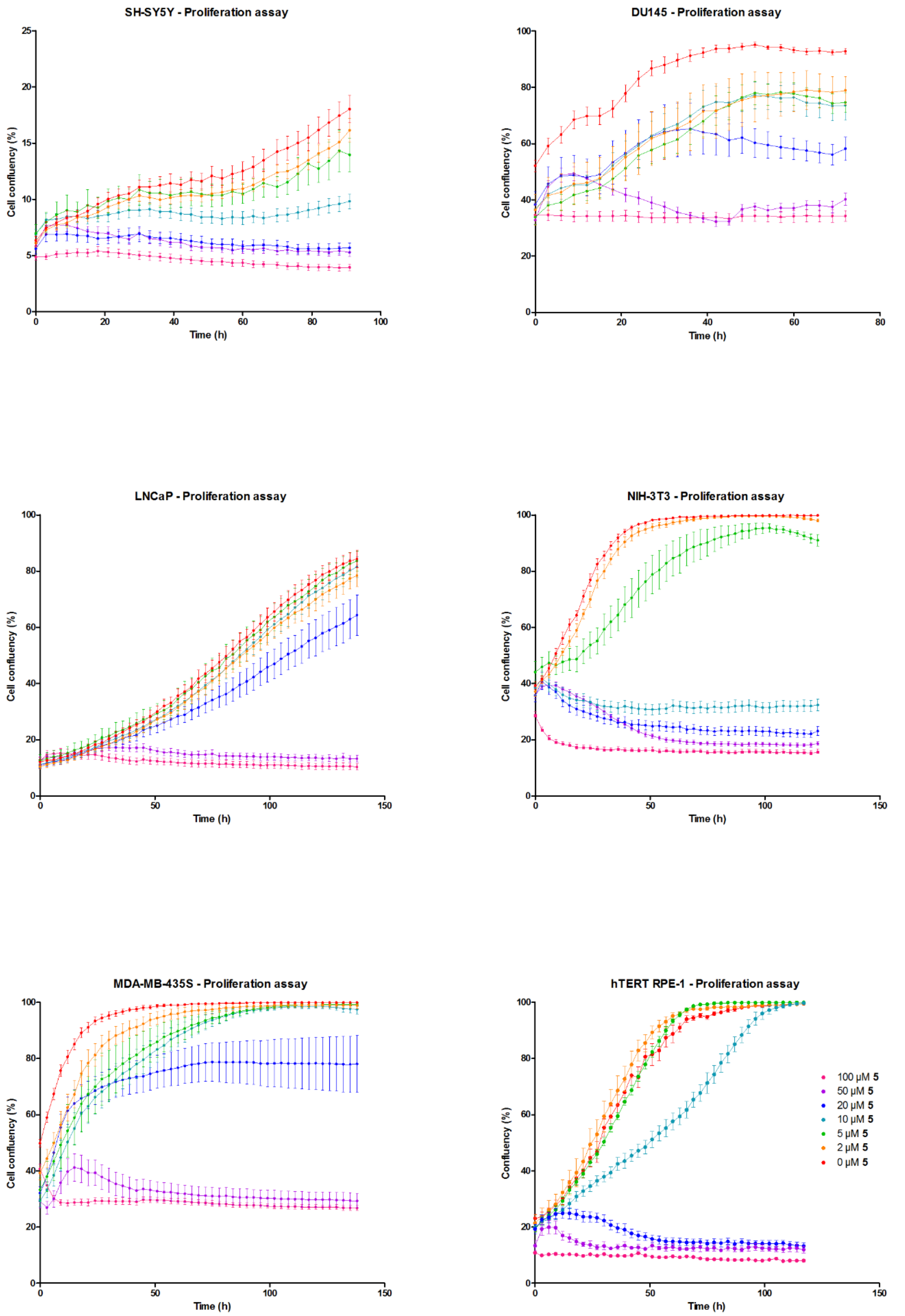

S1 Fig. Investigation of the antiproliferative effect of compound 5 on various cell lines. The pink data points were obtained after addition of 100 µM of **5**, the purple after 50 µM, the dark blue after 20 µM, the light blue after 10 µM, the green after 5 µM, the orange after 2 µM and the red in control situation (n=6).
