# Supplementary figures and images for: Synthesis of novel purpurealidin analogs and evaluation of their effect on the cancer-relevant potassium channel KV10.1
Source: PLoS One. 2017 Dec 8;12(12):e0188811. doi: 10.1371/journal.pone.0188811 (PMC5722316; doi:10.1371/journal.pone.0188811)

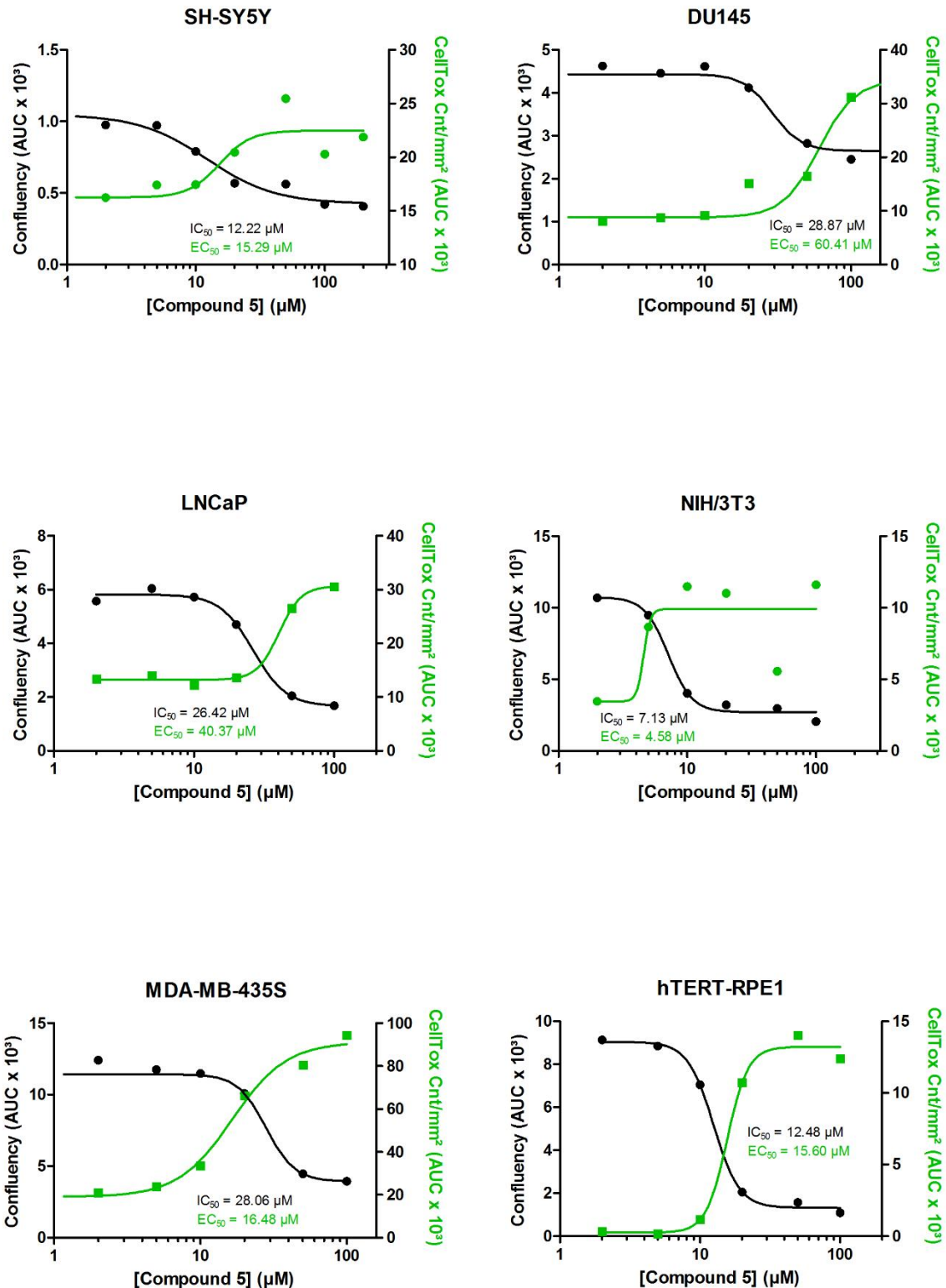

S3 Fig. Antiproliferative and cytotoxic effect of compound 5 on different cell lines.

Supplement: S3 Fig — (PDF) [file pone.0188811.s004.pdf]
