# Supplementary material for: Synthesis of novel purpurealidin analogs and evaluation of their effect on the cancer-relevant potassium channel KV10.1
Source: PLoS One. 2017 Dec 8;12(12):e0188811. doi: 10.1371/journal.pone.0188811 (PMC5722316; doi:10.1371/journal.pone.0188811)
Supplement: S5 Fig — (PDF) [file pone.0188811.s006.pdf]

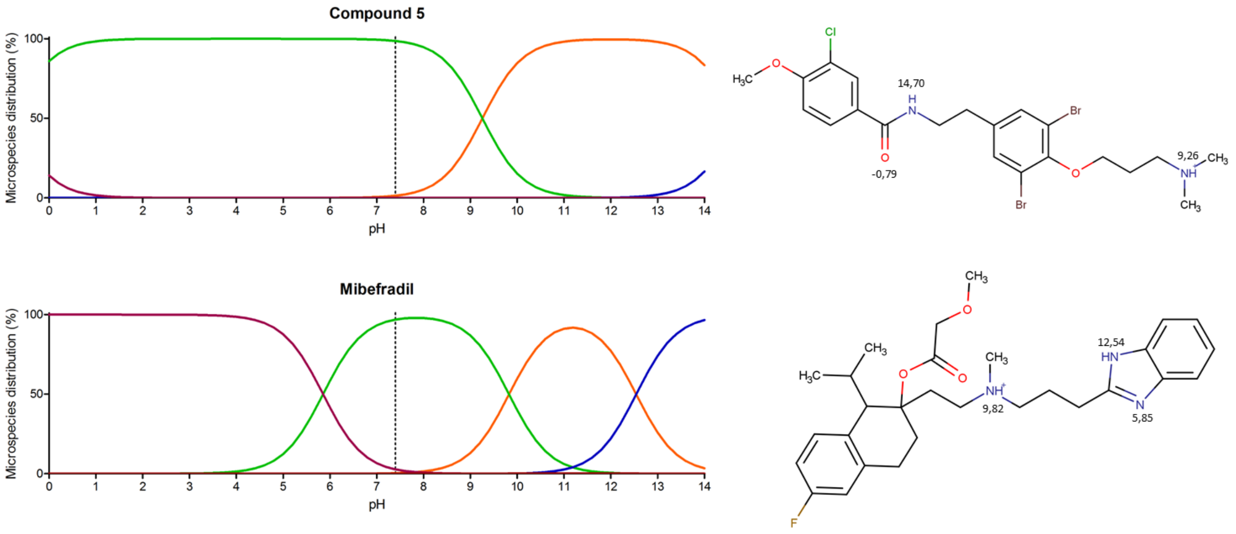

S5 Fig. pK_a based protonation states of compound 5 and mibefradil. The microspecies distribution (%) of the different protonation states of compound **5** and mibefradil was calculated using Marvin (ChemAxon). The dotted line at pH 7.40 (ND96) indicates the major microspecies, which are shown in the right panel. Both compounds carry one positive charge at pH 7.40. p*K*a values were calculated using Marvin and are displayed next to the respective functional groups.
